# Effect of Initiation and Continuous Adherence to ARBs Versus ACEIs on Risk of Adjudicated Mild Cognitive Impairment or Dementia

**DOI:** 10.1093/gerona/glaf028

**Published:** 2025-02-13

**Authors:** Catherine G Derington, Ransmond O Berchie, Daniel O Scharfstein, Ryan M Andrews, Tom H Greene, Yizhe Xu, Jordan B King, Mark A Supiano, Joshua A Sonnen, Jeff Williamson, Nicholas M Pajewski, Jeremy J Pruzin, Jordana B Cohen, Adam P Bress

**Affiliations:** Intermountain Healthcare Department of Population Health Sciences, Spencer Fox Eccles School of Medicine, University of Utah, Salt Lake City, Utah, USA; Intermountain Healthcare Department of Population Health Sciences, Spencer Fox Eccles School of Medicine, University of Utah, Salt Lake City, Utah, USA; Intermountain Healthcare Department of Population Health Sciences, Spencer Fox Eccles School of Medicine, University of Utah, Salt Lake City, Utah, USA; Department of Epidemiology, Boston University School of Public Health, Boston, Massachusetts, USA; Intermountain Healthcare Department of Population Health Sciences, Spencer Fox Eccles School of Medicine, University of Utah, Salt Lake City, Utah, USA; Intermountain Healthcare Department of Population Health Sciences, Spencer Fox Eccles School of Medicine, University of Utah, Salt Lake City, Utah, USA; Intermountain Healthcare Department of Population Health Sciences, Spencer Fox Eccles School of Medicine, University of Utah, Salt Lake City, Utah, USA; Institute for Health Research, Kaiser Permanente Colorado, Aurora, Colorado, USA; Geriatrics Division, Spencer Fox Eccles School of Medicine, University of Utah, Salt Lake City, Utah, USA; University of Utah Center on Aging, Salt Lake City, Utah, USA; Departments of Pathology, Neurology and Neurosurgery, McGill University, Montréal, Québec, Canada; Gerontology and Geriatric Medicine Section, Wake Forest School of Medicine, Winston-Salem, North Carolina, USA; Department of Biostatistics and Data Science, Wake Forest University School of Medicine, Winston-Salem, North Carolina, USA; Banner Alzheimer’s Institute, Phoenix, Arizona, USA; Department of Medicine, Renal-Electrolyte and Hypertension Division, Perelman School of Medicine at the University of Pennsylvania, Philadelphia, Pennsylvania, USA; Department of Biostatistics, Epidemiology, and Informatics, Perelman School of Medicine, University of Pennsylvania, Philadelphia, Pennsylvania, USA; Intermountain Healthcare Department of Population Health Sciences, Spencer Fox Eccles School of Medicine, University of Utah, Salt Lake City, Utah, USA; George E. Wahlen Department of Veterans Affairs Medical Center, Salt Lake City, Utah, USA; (Medical Sciences Section)

**Keywords:** Antihypertensive, Cognition, Hypertension, Medication, Treatment

## Abstract

**Background:**

Whether the differing mechanistic effects between angiotensin-2 receptor blockers (ARBs) and angiotensin-converting enzyme inhibitors (ACEIs) on the renin-angiotensin system translate to differential effects on clinical cognitive outcomes is unclear.

**Methods:**

We employed an active comparator, new-user cohort study to emulate a target trial evaluating the per-protocol effect of initiating and continuously adhering to an ARB versus ACEI on adjudicated amnestic mild cognitive impairment (MCI) and probable dementia (PD) in the Systolic Blood Pressure Intervention Trial (SPRINT). Inverse probability of treatment and censoring weighted cumulative incidence functions accounted for confounding, the competing risk of death, adherence, and loss to follow-up.

**Results:**

Of 9,361 SPRINT participants (mean age 67.1 ± 9.5 years, 36.7% female, 58.7% non-Hispanic White), 710 and 1,289 were new users of an ARB or ACEI. Overall, 291 (41.0%) ARB initiators and 854 (66.3%) ACEI initiators were nonadherent during follow-up. The IP-weighted 4-year probabilities of full adherence and being alive among ARB was 56.0% (95% CI: 52.2%–59.9%) and 30.5% (95% CI: 28.0%–33.1%) for ACEI. The 4-year weighted risk ratios (RR) for amnestic MCI/PD and for amnestic MCI/PD/death with initiation and full adherence to ARB versus ACEI were 0.94 (95% CI: 0.66–1.29) and 0.79 (95% CI: 0.58–1.06). The weighted 4-year weighted RR for all-cause death with ARB versus ACEI initiation and adherence was 0.36 (95% CI: 0.14-0.76).

**Conclusions:**

In this target trial emulation of older adults at high risk for cardiovascular disease, there was insufficient evidence to conclude a beneficial effect of initiating and continuously adhering to an ARB versus ACEI on adjudicated clinical cognitive outcomes.

## Introduction

High blood pressure (BP) is a modifiable risk factor for cognitive decline and dementia (1). Angiotensin-2 receptor blockers (ARB) and angiotensin-converting enzyme inhibitors (ACEI) are first-line treatments for high BP and act on different components of the renin-angiotensin system (RAS), potentially resulting in differential effects on the brain and cognition. ARBs may more effectively prevent cognitive decline and dementia due to their direct antagonism of angiotensin type (AT) 1 receptor, thereby shifting circulating angiotensin-II to bind and activate AT2 and AT4 receptors, reducing oxidative stress, neuroinflammation, and endothelial dysfunction while improving cerebral hypoperfusion (2,3). In contrast, ACEIs lower circulating angiotensin-II levels upstream of where ARBs act in the RAS, thereby reducing angiotensin-II activity on all AT receptors, including any potential beneficial effects derived from stimulating AT2 and AT4.

There are no randomized trials comparing the effects of initiating ARBs versus ACEIs on *adjudicated* clinical cognitive outcomes (eg, dementia or adjudicated mild cognitive impairment [MCI], a precursor to dementia). In the absence of evidence from RCTs, rigorous causal analysis of high-quality observational data can be used to gain insight into the effect of antihypertensive medications on clinical cognitive outcomes. An observational intent-to-treat (ITT) analysis of new users of ARBs versus ACEIs in the Systolic Blood Pressure Intervention Trial (SPRINT) showed lower, but not appreciably different, risks of amnestic mild cognitive impairment (MCI) or dementia over 4.9 years of follow-up (hazard ratio [HR] 0.93 ARB vs ACEI, 95% confidence interval [CI] 0.76-1.13) (4). We also observed a lower risk of all-cause death among ARB versus ACEI initiators (HR 0.48, 95% CI 0.28-0.82). However, ITT analyses can be biased toward the null because they do not estimate the effect of initiating and adhering to treatment during follow-up (5). Modern causal inference methods allow estimation of the “per-protocol effect” (PP): the effect of initiating and adhering to a treatment regimen while minimizing selection bias (6,7).

We used data from SPRINT to estimate the per-protocol effect of initiating and continuously adhering to an ARB- versus ACEI-based antihypertensive medication regimen on MCI and dementia. Our approach aligns with current best practices, including the comparison of clinically realistic treatment strategies, proper handling of time zero, and principled adjustment for baseline and time-varying confounders.

## Method

### Study Design, Setting, and Population

We emulated a target trial in which participants meeting SPRINT eligibility criteria are randomized to initiate and adhere to an ARB- versus ACEI-based antihypertensive regimen. To emulate this trial, we built an observational cohort of patients who initiated ARB- and ACEI-based antihypertensive medication regimens in the first 12 months of SPRINT (Supplementary Figure 1 for schema). The protocol of the hypothetical target trial and the steps for its emulation are shown in **Table 1**.

**Table 1.** Hypothetical Target Trial and Steps for its Emulation.

Component	Hypothetical Target Trial	Emulated Target Trial
Aim	To compare the effect of ARB vs ACEI initiation and continuous adherence on observed outcomes.	Same.
Eligibility	1. Meet SPRINT eligibility criteria 2. No history of ARB or ACEI use prior to baseline 3. Initiate ARB or ACEI within 12 months of SPRINT randomization 4. Non-missing baseline covariates	Same (note that history of baseline ARB or ACEI use is assessed via patient-self report).
Treatment strategies	1. Initiate ARB treatment and remain on it during follow-up according to a clinical protocol 2. Initiate ACEI treatment and remain on it during follow-up according to a clinical protocol	Same.
Treatment assignment	Random assignment to one of the two treatment strategies above.	Participants are assigned to treatment strategies based on study-ascertained use of ARB or ACEI (pill bottle review with pharmacy or medical record validation as available). Randomization is emulated via use of propensity score in baseline treatment model (see Methods).
Follow-up	Participants are followed from randomization until earliest occurrence of: death, loss to follow up (ie, drop-out), or study end date.	Participants are followed from date of treatment initiation until earliest occurrence of: death, loss to follow-up (ie, drop-out), non-terminal event of interest (eg, mild cognitive impairment or probable dementia), or SPRINT study end date.
Outcome	Incidence of clinical diagnosis of mild cognitive impairment, autopsy-confirmed dementia, or death.	In SPRINT, clinical cognitive outcomes (ie, mild cognitive impairment and probable dementia) were identified and adjudicated by a committee (see Methods and Supplemental Methods text). Mild cognitive impairment was further differentiated into amnestic and non-amnestic.
Causal contrast	The effect of initiating and full adherence to ARB vs. ACEI on clinical cognitive outcomes under no loss to follow-up.	Same.
Statistical analysis	Per-protocol analysis	Same, incorporating weights to estimate probabilities of baseline treatment assignment, adherence, and being on-study.

*Notes*: ACEI = angiotensin-converting enzyme inhibitor; ARB = angiotensin receptor blocker; SPRINT = Systolic Blood Pressure Intervention Trial.

Details regarding SPRINT are available elsewhere (8–10). Briefly, between November 2010 and March 2013, U.S. adults ≥ 55 years old at high cardiovascular disease (CVD) risk were randomized 1:1 to target an intensive systolic BP goal of < 120 mm Hg or a standard systolic BP goal of < 140 mm Hg. Key exclusion criteria were diabetes, history of stroke, heart failure, living in a nursing home, diagnosis of dementia, or receiving medications primarily for dementia (**Supplementary Methods**) (11). Investigators adjusted antihypertensive medications according to an evidence-based protocol to the randomized treatment goal (11,12). The SPRINT parent study was approved by the Institutional Review Board at each participating site, and each participant provided written informed consent. The current analysis was approved by the Institutional Review Board at the University of Utah.

Among the 9 361 randomized SPRINT participants, 5 469 were taking an ARB or ACEI at baseline and were excluded from this analysis to remove prevalent users (Supplementary Figure 2). After the removal of these participants, we then identified those who initiated an ARB or an ACEI during the first 12 months after randomization (*N* = 2 040). We also excluded 41 participants with missing baseline covariates (*N* = 17 ARB and *N* = 24 ACEI).

Our final analytic cohort included 710 ARB initiators and 1 289 ACEI initiators (total *N* = 1 999). The date of ARB or ACEI initiation defined the index date (ie, time zero), and we built a long-form, patient-month dataset to track longitudinal changes in medication use, clinical variables, and outcomes post-index (Supplementary Methods).

### Treatment Strategies and Assignment

The treatment strategies were initiating and continuously adhering to (a) an ARB-based antihypertensive medication regimen or (b) an ACEI-based antihypertensive medication regimen. In each follow-up month, we evaluated adherence to an initial treatment protocol of ARB or ACEI when clinically indicated (ie, the participant used the medication when it was clinically appropriate for them to do so) (5). See supplement for how adherence status was determined in each follow-up month based on medication use data and development of contraindications. Factors that defined a contraindication to treatment included abnormal elevations in serum potassium (>5.5 mEq/L); serum creatinine increased by at least 50% to a value of ≥1.5 mg/dL; angioedema, acute kidney injury, or acute renal failure based on serious adverse event [SAE] reports; or treatment-related SAE noted by the SPRINT investigator.

### Cognitive Assessments

 Assessment of cognitive status in SPRINT has been previously described (Supplementary Methods) (4,10,11). Briefly, cognitive assessments were scheduled for baseline, 24- and 48-months follow up, study closeout, and an additional extended follow-up visit (the last available cognitive assessment was July 22, 2018). An expert review panel assessed data collected from standardized cognitive assessments, extended cognitive batteries, and other health assessments (eg, SAE hospitalization reports, depressive scales, quality of life) to categorize participants as: having no cognitive impairment, MCI (sub-classified into amnestic or non-amnestic), probable dementia, or unclassifiable.

### Clinical Cognitive Outcomes

In the SPRINT protocol (11), MCI was defined *a priori* as the last of 2 or more consecutive occurrences of an adjudicated classification of MCI, which we call “protocol-defined” MCI. For our primary outcome, we chose a single occurrence of amnestic MCI to increase the number of events in our analysis given its consistent association with an increased risk of progression to dementia (13). In a secondary analysis, we evaluate the first occurrence of any MCI event (amnestic or non-amnestic) as well as protocol-defined MCI.

### Follow-up

Our data set has follow-up from the index date until the last study visit or death. As we were interested in the effects of continuous adherence, participants were censored during the first month of non-adherence for the current analysis. This per-protocol censor date is not a naturally occurring study event (like loss to follow-up or death) but a methodological choice to restrict follow-up to periods of adherence.

### Hypothetical Treatment Scenarios and Clinical Outcomes of Interest

To draw causal inferences about the effect of ARB versus ACEI treatment on clinical cognitive outcomes and death, we consider 2 hypothetical treatment scenarios. In the first, all patients initiate an ARB-based regimen and are fully adherent; in the second, all patients initiate an ACEI-based regimen and are fully adherent. In both scenarios, participants are fully adherent in each month of follow-up to their assigned medication, there is no loss to follow-up before death, and death is a possible outcome for all patients. All time-to-event outcomes in these scenarios are defined as time to observe the first occurrence of the events of interest.

For each patient and for each hypothetical scenario, we defined the following primary clinical outcomes of interest: (a) time to amnestic MCI or probable dementia; and (b) time to amnestic MCI, probable dementia, or death. We also considered secondary observed outcomes that included the following events alone and in various combinations with each other: amnestic MCI, probable dementia, protocol-defined MCI, and death. For the observed outcomes that do not include death (ie, “non-terminal outcomes”), death may pre-empt the development of the outcome of interest, in which case the event cannot be observed. Thus, we compare the distribution of outcomes (across scenarios) that do not incorporate death, consistent with the sub-distribution function approach to handling competing risks (14). These results should be interpreted jointly with comparisons (across scenarios) of the associated composite outcome that includes death, consistent with the composite outcome approach to competing risks (15).

### Covariates

A list of all baseline and time-varying variables is available in **Supplementary Table 2**. The covariates were selected a priori based on their potential confounding role in (a) the prediction of adherence or being on-study; or (b) the proposed relationship between ACEI versus ARB use and clinical cognitive outcomes (see directed acyclic graph **Supplementary Figure 3**) (4). Because a participant’s index date could occur after randomization in the parent study, to define baseline covariates for the current study, we used information collected before and after the SPRINT randomization visit, aiming to include the most updated measurements where possible. We used information about the following covariates collected at the pre-randomization visit: sociodemographic characteristics (including self-reported race and ethnicity), comorbidities, cognitive function, and concomitant nonantihypertensive medication use. We used the most recent measurement prior to the index date for the following covariates: systolic BP and diastolic BP, laboratory measurements, and non-ARB or -ACEI antihypertensive medication use. Time-varying covariates (updated monthly) included: use of all other antihypertensive classes; systolic BP; body mass index; and serum potassium, creatinine, and glucose; and all cognitive tests (Montreal Cognitive Assessment, Logical Memory form II, Digit Symbol Coding Test). When a new measurement was not observed for a given month, we carried the last observation forward until a new measurement was obtained.

### Statistical Analysis

We fit 2 structural models related to the distribution of clinical outcomes of interest under full adherence to ARB or ACEI and no loss to followup, with splines used to model follow-up time in months from the index date (ie, we do not assume constant hazard or risk across time for any outcome; **Supplementary Methods**). The first is a logistic regression model for the risk of dying (a terminal outcome) at a given month *t*, conditional on still being alive by month *t* − 1. The second is a logistic regression model for the risk of observing a nonterminal outcome at a given month *t*, conditional on still being alive at month *t* and not having experienced that nonterminal outcome by month *t* − 1. These structural models can be used to compute the full adherence sub-distribution function for the nonterminal outcomes as well as the full adherence distribution for the composite outcomes.

The long-form patient-month dataset for estimating the structural model for death (nonterminal event) includes months when an individual is adherent to their initial treatment, alive, and, if relevant, the month of their death (terminal event).

We incorporated inverse probability (IP) weights into the structural models. The weights involve the following conditional probabilities: (a) initial treatment assignment; (b) continuing to adhere to the initial treatment in each follow-up month; and (c) continuing to remain in the study (ie, not lost to follow-up) in each month. In the on-study and adherence models below, we assumed fixed regression coefficients over time. The denominators of the weights include probabilities estimated from the following models (**Supplementary Methods**):

1. Baseline treatment model: Among individuals who initiated either ARB or ACEI, a logistic regression model for the probability of initiating ARB conditional on the baseline covariates (ie, a “propensity score”; **Supplementary Figure 4**).
2. Adherence model (computed separately among ARB and ACEI initiators): A logistic regression model for the probability of adhering to the initial treatment at month t, conditional on being adherent to the protocol through month *t* − , on-study and alive at month *t*, and baseline and time-varying covariates through month *t* − 1. A spline modeled the effect of time.
3. On-study model (computed separately among ARB and ACEI initiators): A logistic regression model for the probability of being on-study at month *t*, conditional on being on study and alive at month *t* − 1, adhering to the initial treatment protocol through month *t* − 1, and baseline and time-varying covariates through month *t* − 1. A spline modeled the effect of time.

The adequacy of these models was evaluated by computing absolute standardized mean differences (ASMD; see Supplementary Methods). The numerators of the weights include unconditional (on baseline and time-varying covariates) probabilities estimated from adherence and on-study models that included only the effect of time.

For each participant, each patient-month *t* in the long-form datasets received a stabilized weight, where the numerator was equal to the multiplication of the cumulative product of their unconditional adherence probabilities and of their on-study probabilities through month *t*. The denominator was equal to the multiplication of their conditional probability of receiving their initial treatment, cumulative product of their conditional adherence probabilities, and their on-study probabilities through month *t* (**Supplementary Figure 5**) (16).

For nonterminal outcomes, we also fit a structural model to relate the clinical outcome of interest to full adherence under initiation with ARB or ACEI and no loss to follow-up, with splines used to model time (**Supplementary Methods**). For each treatment, we specified a structural logistic regression model for remaining adherent at month *t*, conditional on being alive at month *t* and adherent through month *t* − 1. To incorporate the competing risk of death, this structural model was combined with the structural model for death to compute the ITT sub-distribution function for being adherent and alive through month *t* under initiation with ARB or ACEI. The structural model for full adherence was fit using a long-form patient-month dataset that includes months when an individual is adherent to their initial treatment, alive, and, if relevant, the first month of non-adherence. In the **Supplementary Methods**, we detail the sequential randomization assumptions that underlie our causal analyses. We used bootstrap resampling (1 000 iterations) to generate 95% confidence intervals (CI) (17,18). We used the 2.5% and 97.5% percentiles of the bootstrap distribution to compute 95% confidence intervals.

#### Effect estimates

For month 48 (in a scenario without censoring), we estimated: (a) ITT probabilities of being alive and adherent; (b) per-protocol probabilities of dying; (c) per-protocol probabilities of the nonterminal events; and (d) per-protocol probabilities of the composite outcomes of death, MCI, or probable dementia. We report absolute risks and risk ratios (RR) for each outcome comparing ARB versus ACEI. For comparison to the per-protocol probabilities, we also present weighted and unweighted (“crude”) ITT probabilities.

We conducted subgroup analyses in clinically relevant groups by randomization arm (intensive vs standard), age (<75 vs≥75 years), sex, history of cardiovascular disease, and race and ethnicity group (non-Hispanic Black vs all other). All 2-way interactions between each covariate and the binary subgroups were entered into the baseline treatment, adherence, and on-study models and assessed using least absolute shrinkage and selection operator; all 2-way interactions were shrunk to zero (19). Wald tests were used to compare the difference in treatment effects across subgroup strata. We also performed a sensitivity analysis in which we expanded the initiation window to 24 months after the SPRINT randomization visit. All analyses were completed using R v.4.1.2 (R Foundation for Statistical Computing, Vienna, Austria).

## Results

### Participant Characteristics

Among 1 999 participants in our analytic sample, 710 and 1 289 were new users of ARB or ACEI, respectively, within the first 12 months of SPRINT follow-up (unweighted: mean ± standard deviation age was 67.1 ± 9.5 years, 36.7% were female, 58.7% were non-Hispanic White, 30.1% were non-Hispanic Black, and 9.4% were Hispanic). The median [IQR] time between SPRINT randomization and treatment initiation was 0.03 [0.03–2.13] months for ARB and 0.90 [0.03–2.66] months for ACEI. Before weighting, ARB initiators were more likely than ACEI initiators to be female (42.3% vs 33.6%), Hispanic (12.4% vs 7.7%), and use calcium-channel blockers (40.8% vs 31.4%). ARB initiators were less likely than ACEI initiators to be Non-Hispanic White (53.0% vs 61.9%), have only a high school education (12.8% vs 17.8%), have a history of clinical CVD (10.8% vs 15.4%), and take statins (29.7% vs 36.0%; **Table 2**). ARB initiators also had a higher median score on the Digit Symbol Coding Test compared to ACEI initiators (53.0 vs 51.0) and had similar baseline scores on the Montreal Cognitive Assessment (23.2 vs 22.9 points) and the Logical Memory form II (8.4 vs 8.1 points). After IP weighting by the baseline treatment propensity score, covariates were balanced between the groups at baseline (all ASMDs were <0.1; **Supplementary Figure 6**).

**Table 2.** Baseline Characteristics of SPRINT Participants Included in the Current Analysis Before and After IP Weighting

Variable	Before IP Weighting			After IP Weighting		
	ARB	ACEI	ASMD	ARB	ACEI	ASMD
	(*N* = 710)	(*N* = 1,289)				
Demographics						
Age (years)	67.2 (9.7)	67.1 (9.4)	< 0.01	67.1	67.2	< 0.01
Female	300 (42.3)	433 (33.6)	0.18	36.2	36.5	< 0.01
Race and ethnicity^a^						
Hispanic	88 (12.4)	99 (7.7)	0.16	9.4	9.3	< 0.01
Non-Hispanic Black	232 (32.7)	370 (28.7)	0.09	28.8	29.8	0.02
Non-Hispanic White	376 (53.0)	798 (61.9)	0.18	60.0	59.2	0.02
Other	14 (2.0)	22 (1.7)	0.02	1.8	1.8	< 0.01
Education						
Less than high school	64 (9.0)	131 (10.2)	0.04	10.0	9.8	< 0.01
High school graduate only	91 (12.8)	229 (17.8)	0.13	16.0	16.1	< 0.01
Post high school graduate	256 (36.1)	436 (33.8)	0.05	35.1	34.9	< 0.01
College graduate or greater	299 (42.1)	493 (38.2)	0.08	38.9	39.2	< 0.01
Uninsurance	109 (15.4)	191 (14.8)	0.01	14.9	15.0	< 0.01
Usual source of care						
Doctors’ office/outpatient clinic	589 (83.0)	1035 (80.3)	0.07	81.2	81.1	< 0.01
Community healthcare facility/other	79 (11.1)	145 (11.2)	< 0.01	11.0	11.3	0.01
No usual source of care	42 (5.9)	109 (8.5)	0.10	7.8	7.5	0.01
Medical history						
Clinical CVD	77 (10.8)	198 (15.4)	0.13	13.8	13.8	< 0.01
History of atrial fibrillation	54 (7.6)	89 (6.9)	0.03	7.4	7.4	< 0.01
History of depression	134 (18.9)	239 (18.5)	< 0.01	18.2	18.6	0.01
Baseline cognitive assessments						
Montreal cognitive assessment^b^	23.2 (4.0)	22.9 (4.0)	0.08	23.0	23.0	< 0.01
Logical Memory form II^c^	8.4 (3.3)	8.1 (3.3)	0.10	8.2	8.2	< 0.01
Digit symbol coding test^d^	52.6 (15.3)	50.6 (14.6)	0.13	51.1	51.2	< 0.01
Clinical/laboratory measurements						
Systolic BP (mm Hg)	143.4 (15.5)	142.0 (15.4)	0.09	142.4	142.5	< 0.01
Diastolic BP (mm Hg)	79.9 (12.3)	79.2 (12.4)	0.06	79.4	79.4	< 0.01
Resting heart rate (beats/minute)	67.0 (12.2)	67.5 (11.8)	0.04	67.3	67.3	< 0.01
Serum potassium (mg/dL)	4.1 (0.5)	4.1 (0.5)	0.03	4.1	4.1	< 0.01
Serum creatinine (mg/dL)	1.0 (0.3)	1.0 (0.3)	0.01	1.0	1.0	< 0.01
Total cholesterol (mg/dL)	198.6 (40.3)	196.8 (43.2)	0.04	197.9	197.6	< 0.01
HDL cholesterol (mg/dL)	54.6 (14.3)	53.2 (14.7)	0.10	53.5	53.7	0.01
Triglycerides (mg/dL)	124.0 (71.2)	130.1 (103.9)	0.06	129.0	128.3	< 0.01
Body mass index (kg/m^2^)	29.8 (5.8)	29.4 (5.8)	0.08	29.5	29.5	< 0.01
Serum glucose (mg/dL)	98.8 (14.4)	99.0 (13.0)	0.01	98.9	98.8	< 0.01
Medication use						
Aspirin	322 (45.4)	628 (48.7)	0.07	48.0	47.8	< 0.01
Statin	211 (29.7)	464 (36.0)	0.13	34.2	33.9	< 0.01
NSAID	238 (33.5)	433 (33.6)	< 0.01	33.0	33.5	0.01
Number of non-antihypertensive medications	3.2 (2.9)	3.4 (3.1)	0.05	3.3	3.3	< 0.01
Number of antihypertensive medications	2.4 (1.0)	2.3 (0.9)	0.11	2.3	2.3	< 0.01
CCB	290 (40.8)	405 (31.4)	0.20	34.8	34.7	< 0.01
Thiazide diuretic	328 (46.2)	645 (50.0)	0.08	48.5	48.7	< 0.01
Loop diuretic	36 (5.1)	60 (4.7)	0.02	4.8	4.9	< 0.01
Beta-blocker	232 (32.7)	408 (31.7)	0.02	31.6	31.9	< 0.01
Alpha-blocker	32 (4.5)	53 (4.1)	0.02	3.8	4.1	0.01
Other antihypertensive sub-class^e^	58 (8.2)	74 (5.7)	0.10	6.7	6.7	< 0.01
Intensive treatment arm	444 (62.5)	813 (63.1)	0.01	62.9	63.1	< 0.01

*Notes*: ACEI = angiotensin-converting enzyme inhibitor; ARB = angiotensin-II receptor blocker; ASMD = absolute standardized mean difference; CCB = calcium channel blocker; CVD = cardiovascular disease; BP = blood pressure; HDL = high-density lipoprotein; NSAID = non-steroidal anti-inflammatory drug; SPRINT = Systolic Blood Pressure Intervention Trial. Numbers are number (column %), plus–minus values are means ± standard deviation, or square brackets are median [IQR]. The total numbers of patients in the postinverse probability weighted columns are omitted because the numbers are generated from a pseudo population as a result of the weighting.

^a^”Other” includes Asian, Hawaiian or Pacific Islander, Native American, and other as reported by the participant.

^b^Scores range from 0 to 30, with higher scores denoting better cognitive function.

^c^Subtest of the Wechsler Memory scale. Scores range from 0 to 14, with higher scores denoting better cognitive function.

^d^Subtest of the Wechsler Adult Intelligence scale. Scores range from 0 to 135, with higher scores denoting better cognitive function.

^e^Includes centrally-acting agents, vasodilators, potassium-sparing diuretics.

### Adequacy of Adherence and Censoring Models

We evaluated the adequacy of the adherence and censoring models by computing balance with respect to the baseline and time-varying covariates that were included in these models. For each model treatment group and covariate, an ASMD aggregated over months was computed (see **Supplementary Figures 7****–****8**). All ASMDs were <0.1, except for number of nonantihypertensive medications (ASMD = 0.1034) and systolic BP (ASMD = 0.1024) in the ARB adherence model.

### Treatment Adherence

**Figure 1.**
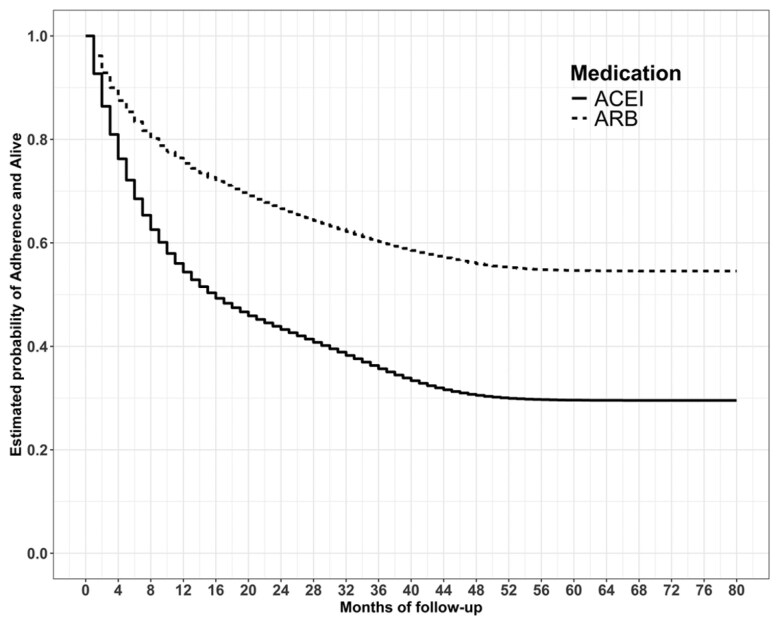
Causal adherence plot estimating the probability of full adherence and being alive by a follow-up month in a scenario without censoring under the two treatments. Abbreviations: ACEI = angiotensin-converting enzyme inhibitor; ARB = angiotensin-II receptor blocker..

Overall, 291 (41.0%) ARB initiators and 854 (66.3%) ACEI initiators were observed to be non-adherent during follow-up. The weighted treatment-specific cumulative incidence curve representing the ITT probabilities of being alive and adherent is shown in **Figure 1**. The weighted 4-year probabilities of full adherence and being alive were 56.0% (95% CI: 52.2%–59.9%) under ARB initiation and 30.5% (95% CI: 28.0%–33.1%) under ACEI initiation. This translates to a risk difference of 25.5% (95% CI: 20.9%–30.2%), indicating better adherence for ARB initiation compared to ACEI initiation.

### Outcomes

**Figure 2.**
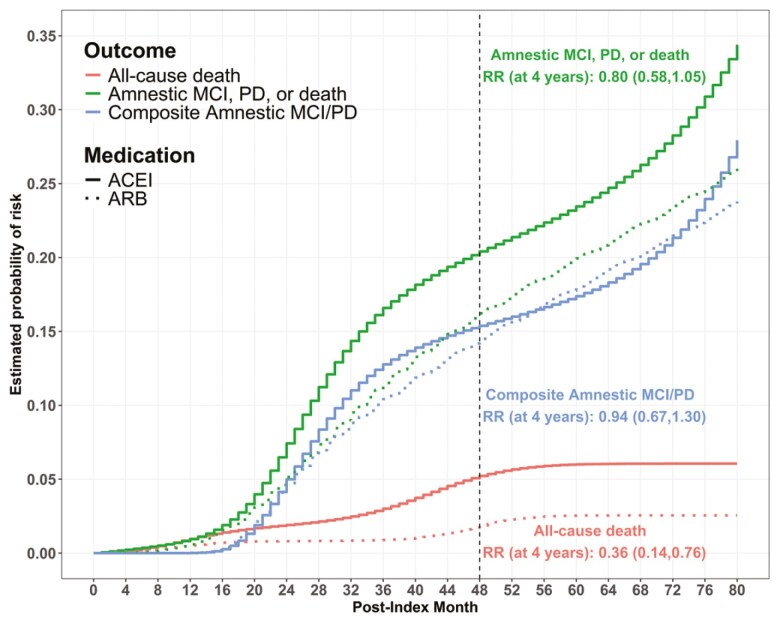
IP-weighted risk curves for the primary composite outcome of amnestic MCI or probable dementia comparing continuous use of ARB vs. ACEI in SPRINT. Abbreviations: ACEI = angiotensin-converting enzyme inhibitor; ARB= angiotensin-II receptor blocker; MCI = mild cognitive impairment; PD = probable dementia; RR = risk ratio; SPRINT = Systolic Blood Pressure Intervention Trial.

For all primary and secondary outcomes, the per-protocol effect estimates favored ARB over ACEI. The treatment-specific cumulative incidence curves representing the estimated per-protocol probabilities of amnestic MCI, probable dementia, or death; amnestic MCI or probable dementia; and all-cause death are shown in **Figure 2**. At 4 years, the per-protocol risk of the primary composite outcome of amnestic MCI or probable dementia was 14% for ARB and 15% for ACEI (RR 0.94; 95% CI: 0.66 to 1.29; **Table 3**). When death was added to this composite, the 4-year per-protocol risk was 16% for ARB and 20% for ACEI (RR 0.79; 95% CI: 0.58–1.06). The 4-year per-protocol risk for all-cause death was 2% for ARB and 5% for ACEI (RR 0.36; 95% CI: 0.20–0.64), indicative of a mortality benefit of ARB versus ACEI. For other secondary non-terminal outcomes, we found inconclusive evidence of a per-protocol effect. However, when rare non-terminal outcomes (probable dementia or protocol-defined MCI) were combined with mortality, a benefit of ARB over ACEI was observed.

**Table 3.** Risks for the Primary and Secondary Outcomes Among Initiation and Continual Use of ARB Versus ACEI

Outcome	Unadjusted (ie, Crude)			IP-Weighted ITT Analysis			IP-Weighted Per-Protocol		
	Absolute Risk at 4 Years (95% CI)		4-Year Risk Ratio (95% CI)	Absolute Risk at 4 Years (95% CI)		4-Year Risk Ratio (95% CI)	Absolute Risk at 4 Years (95% CI)		4-Year Risk Ratio (95% CI)
	ARB (*N* = 710)	ACEI (*N* = 1289)		ARB	ACEI		ARB protocol	ACEI protocol	
Primary outcomes									
Amnestic MCI or probable dementia	0.14 (0.11,0.16)	0.15 (0.13,0.17)	0.88 (0.70,1.10)	0.15 (0.12,0.18)	0.15 (0.13,0.17)	1.01 (0.81,1.24)	0.14 (0.11,0.18)	0.15 (0.12,0.19)	0.94 (0.67,1.30)
Amnestic MCI, probable dementia, or death	0.16 (0.13,0.18)	0.21 (0.19,0.23)	0.75 (0.61,0.92)	0.17 (0.14,0.20)	0.20 (0.18,0.22)	0.86 (0.71,1.03)	0.16 (0.12,0.20)	0.20 (0.17,0.24)	0.80 (0.58,1.05)
Secondary outcomes									
Amnestic MCI alone	0.12 (0.10,0.14)	0.14 (0.12,0.16)	0.86 (0.67,1.10)	0.13 (0.11,0.16)	0.13 (0.12,0.15)	0.98 (0.76,1.23)	0.13 (0.09,0.17)	0.14 (0.11,0.17)	0.95 (0.64,1.35)
Amnestic MCI or death	0.14 (0.12,0.17)	0.19 (0.17,0.21)	0.74 (0.60,0.91)	0.15 (0.13,0.18)	0.18 (0.16,0.21)	0.83 (0.66,1.01)	0.14 (0.11,0.18)	0.18 (0.15,0.22)	0.79 (0.56,1.09)
Any MCI alone^*^	0.14 (0.12,0.18)	0.17 (0.14,0.18)	0.83 (0.76,1.16)	0.15 (0.12,0.18)	0.16 (0.14,0.18)	0.94 (0.76,1.16)	0.15 (0.11,0.20)	0.16 (0.13,0.20)	0.95 (0.68,1.33)
Any MCI^*^ or death	0.16 (0.13,0.19)	0.22 (0.20,0.24)	0.74 (0.60,0.89)	0.17 (0.14,0.20)	0.21 (0.19,0.23)	0.83 (0.68,1.00)	0.17 (0.13,0.22)	0.20 (0.17,0.24)	0.86 (0.64,1.15)
Probable dementia alone	0.02 (0.01,0.03)	0.02 (0.01,0.03)	0.95 (0.45,1.92)	0.02 (0.01,0.04)	0.02 (0.01,0.02)	1.30 (0.58,2.59)	0.02 (0.00,0.03)	0.02 (0.01,0.05)	0.62 (0.14,2.28)
Probable dementia and death	0.04 (0.03,0.06)	0.08 (0.06,0.09)	0.53 (0.33,0.80)	0.04 (0.03,0.06)	0.07 (0.06,0.09)	0.61 (0.38,0.92)	0.03 (0.02,0.05)	0.08 (0.05,0.10)	0.44 (0.22,0.78)
Protocol-defined MCI alone	0.06 (0.04,0.08)	0.08 (0.06,0.09)	0.78 (0.53,1.11)	0.07 (0.05,0.09)	0.07 (0.06,0.09)	0.93 (0.64,1.34)	0.06 (0.03,0.08)	0.07 (0.04,0.09)	0.84 (0.45,1.43)
Protocol-defined MCI or death	0.08 (0.06,0.10)	0.13 (0.11,0.15)	0.63 (0.47,0.83)	0.09 (0.07,0.12)	0.13 (0.11,0.14)	0.72 (0.53,0.96)	0.07 (0.05,0.10)	0.12 (0.09,0.15)	0.61 (0.37,0.92)
Protocol-defined MCI or probable dementia	0.08 (0.06,0.10)	0.09 (0.08,0.11)	0.83 (0.59,1.14)	0.09 (0.06,0.11)	0.09 (0.07,0.10)	1.01 (0.71,1.37)	0.07 (0.05,0.10)	0.09 (0.06,0.11)	0.83 (0.49,1.32)
Protocol-defined MCI, probable dementia, or death	0.10 (0.07,0.12)	0.14 (0.12,0.16)	0.67 (0.51,0.87)	0.11 (0.08,0.14)	0.14 (0.12,0.16)	0.79 (0.60,1.02)	0.09 (0.06,0.12)	0.14 (0.11,0.17)	0.64 (0.42,0.91)
All-cause death	0.02 (0.01,0.03)	0.06 (0.05,0.07)	0.39 (0.21,0.62)	0.02 (0.01,0.03)	0.06 (0.05,0.07)	0.39 (0.20,0.63)	0.02 (0.01,0.03)	0.05 (0.03,0.08)	0.36 (0.14,0.76)

*Notes*: ACEI = angiotensin-converting enzyme inhibitor; ARB = angiotensin-II receptor blocker; CI = confidence interval; IP = inverse probability; ITT = intention-to-treat; MCI = mild cognitive impairment.

^*^amnestic or non-amnestic.

### Subgroup and Sensitivity Analyses

With the exception of history of CVD, there was insufficient evidence to conclude that treatment estimates for amnestic MCI, probable dementia, or death, amnestic MCI or probable dementia, and all-cause death, varied by levels of the subgroups (**Supplementary Table 4**). For amnestic MCI or probable dementia, the per-protocol 4-year RR was 0.46 (95% CI: 0.17–1.00) for those with a history of CVD and 1.09 (95% CI: 0.74–1.60) for those without a history of CVD (interaction *p*-value = .04). For amnestic MCI, probable dementia, or death, these per-protocol 4-year RR were 0.44 (95% CI: 0.21–0.81) and 0.91 (95% CI: 0.63 to 1.26; interaction *p*-value = .04), respectively. For all-cause mortality, the risks in 2 subgroups were comparable (interaction *p*-value = .91).

In sensitivity analyses, results were similar to the main analysis after expanding the initiation window to 24 months post-SPRINT randomization and when treating SAEs as a protocol deviation (**Supplementary Table 5**).

## Discussion

In this target trial emulation using data from SPRINT, there was insufficient evidence to conclude a beneficial effect of continuous adherence to ARBs over ACEIs on adjudicated clinical cognitive outcomes among U.S. adults at high CVD risk undergoing treatment for high BP. The direction of effect was consistent with the hypothesis that ARBs are associated with a lower risk of clinical cognitive outcomes versus ACEIs, but uncertainty around effect estimates was high. The hypothesis is based on animal, human, and population data suggesting that medications that stimulate (eg, ARBs) versus inhibit (eg, ACEIs) AT2/AT4 receptors are associated with greater beneficial effects on the brain (20–23). In the current study, ACEI initiators were significantly more likely to discontinue treatment than ARB initiators, consistent with data indicating that ARBs are better tolerated than ACEIs (eg, bradykinin cough associated with ACEI use occurs in 3%–30% of patients) (24). We did find a large effect of initiating and adhering to an ARB versus ACEI on all-cause death, which is difficult to explain, as prior studies have not shown such an effect (25,26). Studies with larger sample sizes, longer follow-up times, and more clinical cognitive events are needed to more precisely estimate per-protocol effects of initiating and continuously adhering to ARB- versus ACEI-based antihypertensive medication regimens.

To the best of our knowledge, this is the first analysis to estimate the per-protocol effect of initiating and continuously adhering to an ARB- versus ACEI-based antihypertensive regimen and clinical cognitive outcomes using modern causal inference approaches and target trial emulation techniques. Per-protocol effects are important to consider because ITT estimands can mask treatment effects in the presence of non-adherence, potentially making harmful medications appear safe or effective treatments appear ineffective (5). This is important because the potential effect of these drugs most likely takes a substantial amount of time to manifest. Our prior ITT analysis of SPRINT found similar results, showing a lower but not appreciably different risk of amnestic MCI or dementia over 4.9 years of follow-up (hazard ratio 0.93 for ARB versus ACEI, 95% CI: 0.76–1.13) (4). We hypothesized that the per-protocol analysis would show an augmented beneficial effect of ARB versus ACEI initiation and continuous use. The substantial uncertainty in our effect estimates may be, in part, from participants being removed from the risk set over time because non-adherence is added as an additional censoring mechanism in the per-protocol analysis, leading to decreased sample size over time.

Animal and mechanistic data show that ARBs work in different places in the renin-angiotensin system (RAS) and have greater neuroprotective effects than ACEIs (20,27–32). Both ACEIs and ARBs inhibit the activation of AT1 receptors and the ACE/Ang II/AT1 receptor axis. However, ARBs might have more pronounced effects on the brain because they selectively and competitively block AT1 receptor, preventing Ang II from binding to AT1 receptors while allowing and even upregulating Ang II to bind to the neuroprotective receptors AT2 receptor and AT4 receptor. By directly binding AT1 receptors, ARBs increase Ang II production due to negative feedback regulation, resulting in direct stimulation of AT2 receptors and cleavage of Ang II into Ang IV, which enhances the activity of AT4 rececptors (20,27,32–34). Conversely, ACEIs reduce ACE activity, leading to decreased downstream Ang II production and its interaction with all Ang receptors. The elevated circulating Ang II levels with ARB treatment promote the unopposed activation of the Ang II/AT2/4 receptor pathways, which are associated with various neurocognitive benefits including improved cerebral perfusion and brain metabolism, as well as maintaining brain homeostasis by reducing hypoxia, inflammation, oxidative stress, and cellular apoptosis. Additionally, it is reasonable to assume that declining cognitive function may impair one’s ability to manage treatment, thereby contributing to nonadherence. Although our inverse probability weighting approach adjusts for time-varying cognitive measures, residual confounding due to unmeasured aspects of cognitive impairment could bias results toward the null, potentially underestimating the benefits of initiating and adhering to ARBs relative to ACEIs.

Our prior observational studies and several other recent ones (35–38) suggest that antihypertensive medication regimens that exclusively stimulate versus inhibit AT2/AT4 receptor, driven by differences in ARB (stimulating) and ACEI (inhibiting) use, are associated with lower rates of MCI and Alzheimer’s disease (AD) and AD-related dementias (AD/ADRD). In a recent meta-analysis of 15 observational studies and 7 RCTs, treatment with ARBs was associated with lower dementia risk than treatment with other antihypertensives (39). In another secondary analysis of SPRINT data, we found that prevalent use of AT2/AT4 receptor-stimulating versus inhibiting regimens was associated with a 25% lower risk of adjudicated MCI or probable dementia during 4.8 years of follow-up (36). In a follow-up study of Medicare beneficiaries, new use of AT2/AT4 receptor-stimulating versus inhibiting regimens was associated with a 16% lower risk of ADRD during a median of 6.9 years (38). A recent analysis of three Dutch General Practice Registration Networks involving 133 355 patients found that ARBs, CCBs, and other AT2/AT4 receptor-stimulating versus inhibiting antihypertensive medications were associated with lower dementia risk (40). This study extends our prior work by estimating the per-protocol effect of ARBs versus ACEIs, providing a clinically relevant measure of treatment benefits under continuous adherence—a critical consideration in older adults where nonadherence is common, and differential adherence to ARBs versus ACEIs has been previously reported (41). Although the findings are directionally consistent with the ITT analysis, the per-protocol approach highlights the impact of continuous adherence on outcomes and strengthens confidence in the robustness of the results. These insights are particularly relevant to gerontology, where treatment adherence plays a key role in managing cognitive and mortality risks. By accounting for differences in non-adherence between ARB and ACEI initiators using weights, our results offer a robust estimate of the benefits of ARBs vs. ACEIs under continuous adherence.

The strengths of our study include the use of the SPRINT dataset, which included a high-quality assessment of medication use (through pill bottle review and interviews by study investigators) and adjudicated cognitive clinical outcomes. The current study is also strengthened by the active comparator, new user design, which reduces the risk of confounding by indication, as both ARBs and ACEIs are interchangeably recommended first-line treatments for hypertension and cardiovascular risk reduction (42). Additionally, the use of IP weighting effectively balanced baseline and time-varying covariates, minimizing the impact of confounding by indication. The current analysis also incorporates the competing risk of death and does not assume proportional hazards in our structural models. Use of proportional hazards models for analyses with time-varying covariates can lead to biased estimates and incorrect inferences due to potential violations of the proportional hazards assumption, which contradicts the concept of time-varying covariates by asserting that hazards remain constant between groups over time (43). The current analysis incorporated flexible structural models, which have several advantages over traditional models that incorporate proportional hazards: (a) incorporate time-varying treatment effects due to differential adherence patterns; (b) properly handle competing risks such as death; (c) adjust for complex nonlinear relationships among the exposures, confounders, outcomes, censoring, and time; (d) consider the informativeness of censoring; and (e) estimate absolute risks, which are more clinically interpretable for decision-makers. Our findings should be interpreted within the context of the following limitations. First, the SPRINT baseline data (ie, pre-randomization) only include self-reported current use, and there are no data on complete treatment history before current use. Thus, the definition of initiators in our study may include both first-time users and re-initiators (ie, those who took an ACEI or an ARB previously but did not report taking it at the time of the baseline visit). Interpretation of our results is limited to individuals who would have been SPRINT-eligible, and results may differ among individuals who would not have been SPRINT-eligible (ie, those with diabetes or a history of stroke). Second, the relatively high rate of both ARB and ACEI discontinuation likely contributed to lower overall event rates in both groups and the substantial uncertainty around the effect estimates. Because our sample size and event rate were relatively low and we calculated relative risks rather than hazard ratios, we had limited power to detect effects that are reflected in the width of our confidence intervals. Finally, we cannot exclude the possibility that the observed effect estimates were affected by unmeasured confounding.

### Conclusion

In this target trial emulation study of high-CVD-risk US adults, there was insufficient evidence to conclude a beneficial effect of initiation and adherence to ARBs versus ACEIs on the combined risk of amnestic MCI or probable dementia. The directions of effect were consistent with the prior hypothesis that ARBs have a greater beneficial effect on clinical cognitive outcomes than ACEIs. Additional studies with larger sample sizes and longer duration of follow-up are needed to further elucidate whether ARBs should be preferred over ACEIs to delay the onset of cognitive decline among patients receiving treatment for hypertension.

## Supplementary Material

glaf028_suppl_Supplementary_Materials

## Notes

The funding sources had no role in the design and conduct of the study; collection, management, analysis, and interpretation of the data; preparation, review, or approval of the manuscript; and decision to submit the manuscript for publication.
