# Supplementary material for: Effect of Initiation and Continuous Adherence to ARBs Versus ACEIs on Risk of Adjudicated Mild Cognitive Impairment or Dementia
Source: J Gerontol A Biol Sci Med Sci. 2025 Feb 13;80(7):glaf028. doi: 10.1093/gerona/glaf028 (PMC12287627; doi:10.1093/gerona/glaf028)
Supplement: glaf028_suppl_Supplementary_Materials [file glaf028_suppl_supplementary_materials.docx]

Supplemental Content

**Effect of initiation and continuous adherence to ARBs vs. ACEIs on risk of adjudicated mild cognitive impairment or dementia.**

Catherine G. Derington, PharmD, MS;^1^* Ransmond O. Berchie., MS;^1^* Daniel O. Scharfstein, ScD;^1^ Ryan Andrews, PhD, MHS;^2,3^ Tom H. Greene, PhD;^1^ Yizhe Xu, PhD;^1^ Jordan B. King, PharmD, MS;^1,4^ Mark A. Supiano, MD;^5^ Joshua A. Sonnen, MD;^6^ Jeff Williamson, MD;^7^ Nicholas M. Pajewski, PhD;^8^ Jeremy Pruzin, MD,^9^ Jordana B. Cohen, MD, MSCE;^10,11^ and Adam P. Bress, PharmD, MS^1,12^

**Equal contributions*

1. Intermountain Healthcare Department of Population Health Sciences, Spencer Fox Eccles School of Medicine, University of Utah, Salt Lake City, UT
2. Department of Epidemiology, Boston University School of Public Health, Boston, MA
3. Department of Biometry and Data Science, Leibniz Institute for Prevention Research and Epidemiology—BIPS, Bremen, Germany
4. Institute for Health Research, Kaiser Permanente Colorado, Aurora, CO
5. Geriatrics Division, Spencer Fox Eccles School of Medicine, University of Utah, Salt Lake City, UT
6. Departments of Pathology, Neurology and Neurosurgery, McGill University, Montréal, Québec, Canada
7. Gerontology and Geriatric Medicine Section, Wake Forest School of Medicine, Winston-Salem, NC
8. Department of Biostatistics and Data Science, Wake Forest School of Medicine, Winston-Salem, NC
9. Banner Alzheimer’s Institute, Phoenix, AZ
10. Department of Medicine, Renal-Electrolyte and Hypertension Division, Perelman School of Medicine at the University of Pennsylvania, Philadelphia, PA
11. Department of Biostatistics, Epidemiology, and Informatics, Perelman School of Medicine, University of Pennsylvania, Philadelphia, PA
12. George E. Wahlen Department of Veterans Affairs Medical Center, Salt Lake City, UT

[eMethods 3](#_Toc175315952)

[Dataset creation and longitudinal assessment of variables 3](#_Toc175315953)

[Example dataset. 3](#_Toc175315954)

[Example timeline. 3](#_Toc175315955)

[Assessment of cognitive outcomes in SPRINT 3](#_Toc175315956)

[Step 1. 4](#_Toc175315957)

[Step 2. 4](#_Toc175315958)

[Step 3. 4](#_Toc175315959)

[Treatment protocol 5](#_Toc175315960)

[Example Dataset 5](#_Toc175315961)

[Covariate assumptions 6](#_Toc175315962)

[Estimation of weights and parameters of structural models 6](#_Toc175315963)

[eTables 8](#_Toc175315964)

[eTable 1. STROBE Reporting Checklist for Cohort Studies. 8](#_Toc175315965)

[eTable 2. Covariates used for the statistical models. 10](#_Toc175315966)

[eTable 3. Reasons for censoring at each timepoint. 12](#_Toc175315967)

[eTable 4. Primary composite outcome results among subgroups. 13](#_Toc175315968)

[eTable 5. Sensitivity analyses on the primary outcome. 16](#_Toc175315969)

[eFigures 17](#_Toc175315970)

[eFigure 1. Study design schema. 17](#_Toc175315971)

[eFigure 2. Flowchart for inclusion in the current study. 18](#_Toc175315972)

[eFigure 3. Directed acyclic graph describing the proposed relationships between the exposure, covariates, and outcomes. 19](#_Toc175315973)

[eFigure 4. Distribution of probability of baseline ARB initiation among ARB initiators (bottom panel) and ACEI initiators (top panel). 20](#_Toc175315974)

[eFigure 5. Distribution of stabilized inverse probability weights for ARB vs. ACEI treatment across follow-up. 22](#_Toc175315975)

[eFigure 6. Balance of patient characteristics before and after inverse propensity score weighting at baseline. 23](#_Toc175315976)

[eFigure 7. Balance of patient characteristics before and after applying time-varying adherence weights within ARB initiators (Panel A) and ACEI initiators (Panel B). 24](#_Toc175315977)

[eFigure 8. Balance of patient characteristics before and after applying time-varying censoring weights within ARB initiators (Panel A) and ACEI initiators (Panel B). 26](#_Toc175315978)

# Supplemental Methods

Dataset creation and longitudinal assessment of variables
To evaluate time-varying exposure and covariates for each participant, a long dataset was created. Each participant had several rows of data. Each row represents each follow-up month for that participant. Columns included baseline data (unchanged for each row) and time-updated data for each month.

### Example dataset.

| Study ID | Month | BL_age | FU_age | BL_exposure | BL_  dose | FU_exposure | FU _  Dose | BL_SBP | FU_SBP | BL_CVD | FU_CVD |
| --- | --- | --- | --- | --- | --- | --- | --- | --- | --- | --- | --- |
| 1 | 0 | 65 | . | 0 | 20 | . | . | 150 | . | 0 | . |
| 1 | 1 | 65 | 65 | 0 | 20 | 0 | 20 | 150 | 142 | 0 | 0 |
| 1 | 2 | 65 | 65 | 0 | 20 | 0 | 20 | 150 | 142 | 0 | 0 |
| 1 | 3 | 65 | 65 | 0 | 20 | 0 | 20 | 150 | 140 | 0 | 0 |
| 1 | 4 | 65 | 66 | 0 | 20 | 0 | 20 | 150 | 140 | 0 | 0 |
| 1 | 5 | 65 | 66 | 0 | 20 | 0 | 20 | 150 | 140 | 0 | 0 |
| 1 | 6 | 65 | 66 | 0 | 20 | 0 | 20 | 150 | 148 | 0 | 0 |
| 1 | 7 | 65 | 66 | 0 | 20 | 0 | 20 | 150 | 144 | 0 | 0 |
| 1 | 8 | 65 | 66 | 0 | 20 | 0 | 40 | 150 | 144 | 0 | 1 |
| 1 | 9 | 65 | 66 | 0 | 20 | 0 | 40 | 150 | 135 | 0 | 0 |
| 1 | 10 | 65 | 66 | 0 | 20 | 0 | 40 | 150 | 135 | 0 | 0 |
| 1 | 11 | 65 | 66 | 0 | 20 | 1 | 50 | 150 | 135 | 0 | 0 |
| 1 | 12 | 65 | 66 | 0 | 20 | 1 | 100 | 150 | 130 | 0 | 0 |

In SPRINT, antihypertensive medication data and systolic BP values were updated at each treatment visit, which occurred monthly for the first three months after randomization, then every three months for the duration of the trial. Intensive arm participants could be seen more often for *pro re nata* (PRN; “as needed”) treatment visits if needed to titrate antihypertensive medication therapy. The frequency of data collection for covariates is shown in Table 5.1 of the SPRINT study protocol.^1^ Given the monthly follow-up frequency used in the current study, the occurrence of SPRINT visits in which data were collected may have occurred between months in the data. Therefore, time-updated data points reflect the value of the variable if it was updated within the prior month (e.g., month 2 systolic BP values represent the most recent value observed between months 1 and 2). If no new measurement was observed, the last observation is carried forward.

### Example timeline.

## Assessment of cognitive outcomes in SPRINT

SPRINT investigators used 3 steps to ascertain cognitive status at pre-randomization and at the 24-month, 48-month, close-out visit, and extended follow-up visits.

### Step 1.

Trained examiners administered in-person cognitive screening assessments to all participants. Assessments included the Montreal Cognitive Assessment (MoCA) to assess global cognitive function (range 0-30; higher scores indicate better function), the Logical Memory forms I and II subtests of the Wechsler Memory Scale to assess learning and memory (ranges, 0-28 and 0-14, higher scores indicate better function), and the Digit Symbol Coding Test of the Wechsler Adult Intelligence Scale to assess processing speed (range, 0-135, higher scores indicate better function). A preidentified proxy was administered the Functional Activities Questionnaire, a 10-item measure of functional abilities (range, 0-30, higher scores indicate better function) for the following persons and scores: 1) white participant scored lower than 19 (with <12 years of education) or lower than 21 (with ≥12 years of education) on the MoCA; 2) non-white participant scored lower than 17 (with <12 years of education) or lower than 19 (with ≥12 years of education) on the MoCA; 3) any participant with a decrease of ≥5 points from a previous MoCA assessment.

### Step 2.

Only participants who scored >0 on the Functional Activities Questionnaire or ≤1 on the 5-point Delayed Recall subtest of the MoCA underwent an extended cognitive battery to measure attention/concentration, verbal and nonverbal memory, language, and executive functions. Validated telephone batteries were administered for participants who could not attend in-person follow-up visits. For participants receiving the telephone battery, the Functional Activities Questionnaire was administered if the participant scored below a preset cut point (≤31) on the Modified Telephone Interview for Cognitive Status. If a participant had died or was otherwise unable to communicate by telephone, the Dementia Questionnaire was administered to a prespecified contact. For all tests and questionnaires, validated Spanish translations were used when available. Otherwise, instruments were translated and then back-translated.

### Step 3.

An expert adjudication panel that included a neurologist, neuropsychologists, geriatricians, and geropsychologists adjudicated cognitive status, all of whom were masked to treatment group assignment (intensive vs. standard). The panel reviewed data from cognitive test scores and proxy status reports collected in steps 1 and 2 in addition to other data collected as a part of the clinical trial (standardized measurements of depressive symptoms, perceived health status, quality of life, current medications, medical problems, and current health habits [smoking, alcohol use, and physical activity]). The panel also reviewed serious adverse event reports from hospitalizations (treatment group references redacted).

Participants were classified into 1 of 3 primary categories: no cognitive impairment, mild cognitive impairment (MCI), or probable dementia. Unclassifiable cases were placed in a “cannot classify” category. Each case was reviewed independently by 2 adjudicators using standardized diagnostic criteria for probable dementia and MCI. Agreements by the 2 adjudicators were final. Disagreements were discussed by the full panel on regularly scheduled conference calls, with the classification decision achieved by a majority vote of the panel members. MCI was subtyped into amnestic vs. non-amnestic MCI based using criteria adapted from Winblad et al.^2^ A formal categorization of MCI was made upon the second of two consecutive classifications of amnestic or non-amnestic MCI. No subclassification of probable dementia was made. Additional details of the adjudication process can be found in the trial protocol.^1^

## Treatment protocol

If a patient discontinued their initial treatment and a contraindication to treatment occurred in the prior month, they remained in the risk set as adherent to protocol. Contraindications to ARB or ACEI treatment included: abnormal elevations in serum potassium (>5.5 mEq/L); serum creatinine increased by at least 50% to a value of ≥1.5 mg/dL; angioedema, acute kidney injury, or acute renal failure based on serious adverse event [SAE] reports; or treatment-related SAE noted by the investigator). Discontinuation of treatment due to SAEs reasonably expected to be related to treatment (e.g., angioedema) were not considered a protocol deviation as treatment-related SAEs would clinically warrant discontinuation of therapy.^5^ We did not include hypotension or therapeutic inertia (i.e., continuing treatment despite ineffectiveness) in the protocol definition because SPRINT dictated treatment escalation or de-escalation to specific BP ranges, and therapeutic inertia was high overall.^13^

### Example Dataset

| **Patient** | **Month** | **Taking Medication** | **Adherence Status** | **Contraindication** | **Adherence Status with Contraindication** |
| --- | --- | --- | --- | --- | --- |
| 1 | 1 | Yes | Yes | No | Yes |
| 1 | 2 | Yes | Yes | No | Yes |
| 1 | 3 | Yes | Yes | No | Yes |
| 1 | 4 | No | No | No | No |
|  |  |  |  |  |  |
| 2 | 1 | Yes | Yes | No | Yes |
| 2 | 2 | Yes | Yes | No | Yes |
| 2 | 3 | Yes | Yes | Yes | Yes |
| 2 | 4 | No | No | No | Yes (had contraindication at month 3) |
| 2 | 5 | No | No | No | No |
|  |  |  |  |  |  |
| 3 | 1 | Yes | Yes | No | Yes |
| 3 | 2 | Yes | Yes | No | Yes |
| 3 | 3 | Yes | Yes | No | Yes |
| 3 | 4 | No | No | Yes | Yes (has contraindication at month 4) |
| 3 | 5 | Yes | Yes | No | Yes |

Patient 1 stayed on the medication until month 4 where they did not take the medication. This patient is not adherent at month 4 under both definitions of adherence since they did not have any contraindication at either month 3 or 4.

Patient 2 stayed on the medication until month 4 where they did not take the medication. This patient is designated as adherent at month 4 in the primary analysis since they had a contraindication at month 3. However, they become non-adherent at month 5 since they are still not taking the medication.

Patient 3 stayed on the medication until month 4 where they did not take the medication. This patient is designated as adherent at month 4 in the primary analysis since they had a contraindication at month 4. They however take the medication again at month 5 which makes them adherent at month 5 too.

## Covariate assumptions

In our analysis, we make the following assumptions. Lists of covariates are available in eTable 2.

1. Among those who initiated ARB or ACEI during the first year of the parent trial, the decision to initiate ARB vs. ACEI depends only on the baseline covariates.
2. Among those on-study at month t-1, alive at month t and adherent to ARB (or ACEI) through month t-1, adherence to ARB (or ACEI) at month t depends only on the baseline and time-varying covariates through month t-1.
3. Among those on-study and alive at month t-1 and adherent to ARB (or ACEI) through month t-1, the decision to go off-study at month t depends only on the baseline and time-varying covariates through month t-1.

## Estimation of weights and parameters of structural models

In our analysis, we fit two structural models related to the distribution of potential outcomes:

1. (S1) A logistic regression model for the risk of dying under full adherence to ARB (ACEI) conditional on being alive at month t-1 under full adherence to ARB (ACEI); a spline is used to model time.
2. (S2) A logistic regression model for the risk of observing the non-death event under full adherence to ARB (ACEI) conditional on being alive at month t and not having experienced the non-terminal event by month t-1 under full adherence to ARB (ACEI); a spline is used to model time.

Knowledge of the parameters of the structural models can be used to learn the full adherence sub-distribution function for the non-terminal outcomes as well the full adherence distribution for the composite outcomes.

In addition, we fit three additional models:

1. (M1) Among individuals who initiated ARB or ACEI during the first year of the parent trial, we fit a logistic regression model for the probability of initiating ARB conditional on the baseline covariates listed eTable 2.
2. (M2) Among individuals who initiated ARB (ACEI), we fit a logistic regression model for the probability of taking ARB (ACEI) at month t after index, conditional on taking ARB (ACEI) through month t-1, being on-study (i.e., not lost to follow-up) and alive at month t and the baseline/time-varying covariates listed in eTable 2; a spline is used to model time
3. (M3) Among individuals who initiated ARB (ACEI), we fit a logistic regression model for the probability of being on-study at month t after index, conditional on being on-study and alive at month t-1, taking ARB (ACEI) through month t-1 and the baseline and time-varying covariates listed in eTable 2; a spline is used to model time.

For each participant i, we used the fit of model (M1) to estimate the probability of initiating ARB (ACEI) conditional on their baseline covariate ($p_{1,i}$). For each participant i who initiated and adhered to ARB (ACEI) through month t-1 and is on-study and alive at month t, we used the fit of model (M2) to estimate the probability of adhering to ARB (ACEI) at month t conditional on their baseline covariates and time-varying covariates through month t-1 ($p_{2,t,i}$). For each participant i who initiated and adhered to ARB (ACEI) through month t-1 and is on-study and alive at month t-1, we used the fit of model (M3) to estimate the probability of being on-study at month t conditional on their baseline covariates and time-varying covariates through month t-1 ($p_{3,t,i})$. We also fit versions of these models without any person-specific covariates to obtain corresponding probabilities $q_{1},q_{2,t}$ and $q_{3,t}.$

The parameters of models S1 and S2 are estimated by analyzing “long-form” datasets using weighted logistic regression. In the long-form dataset for model (S1), a row is included for each month t that participant i who initiated ARB (ACEI) is alive at time t-1, on-study at month t and adherent to ARB (ACEI) through month t-1; the outcome is an indicator of whether the individual died in month t and the covariates are the spline terms at month t; and the weight is the product of $q_{1/}p_{1,i},q_{2,1/} p_{2,1,i}, \ldots,{q_{2,t-1/}p}_{2,t-1,i},{q_{3,1/}p}_{3,1,i},\ldots,{q_{3,t/}p}_{3,t,i}$. In the long-form dataset for model (S2), a row is included for each month t that participant i who initiated ARB (ACEI) is alive and on-study at time t, free of the non-death event by month t-1 and adherent to ARB (ACEI) through month t; the outcome is an indicator of whether the individual has the non-death event in month t and the covariates are the spline terms at month t; and the weight is the product of ${q_{1/}p}_{1,i}, {q_{2,1/}p}_{2,1,i}, \ldots,{q_{2,t/}p}_{2,t,i},q_{3,1/}p_{3,1,i},\ldots,{q_{3,t/}p}_{3,t,i}$.

For each participant, the inverse of each model was taken to generate a final weight, which was then stabilized. We did not truncate weights because doing so did not yield greater balance in baseline characteristics than the untruncated weights.

# eTables

## eTable 1. STROBE Reporting Checklist for Cohort Studies.

|  | Item No | Recommendation | Page No |
| --- | --- | --- | --- |
| **Title and abstract** | 1 | (*a*) Indicate the study’s design with a commonly used term in the title or the abstract | 1, 3 |
|  |  | (*b*) Provide in the abstract an informative and balanced summary of what was done and what was found | 3-4 |
| Introduction | | | |
| Background/rationale | 2 | Explain the scientific background and rationale for the investigation being reported | 5-6 |
| Objectives | 3 | State specific objectives, including any prespecified hypotheses | 6 |
| Methods | | | |
| Study design | 4 | Present key elements of study design early in the paper | 6  eFigure 1  eMethods |
| Setting | 5 | Describe the setting, locations, and relevant dates, including periods of recruitment, exposure, follow-up, and data collection | 6, 8  eMethods |
| Participants | 6 | (*a*) Give the eligibility criteria, and the sources and methods of selection of participants. Describe methods of follow-up | 6-7 |
|  |  | (*b*) For matched studies, give matching criteria and number of exposed and unexposed | n/a |
| Variables | 7 | Clearly define all outcomes, exposures, predictors, potential confounders, and effect modifiers. Give diagnostic criteria, if applicable | 7-9  eMethods |
| Data sources/ measurement | 8* | For each variable of interest, give sources of data and details of methods of assessment (measurement). Describe comparability of assessment methods if there is more than one group | 7-9 |
| Bias | 9 | Describe any efforts to address potential sources of bias | 7-9 eFigure 3 eMethods |
| Study size | 10 | Explain how the study size was arrived at | 7, eFigure 2 |
| Quantitative variables | 11 | Explain how quantitative variables were handled in the analyses. If applicable, describe which groupings were chosen and why | 8-9 |
| Statistical methods | 12 | (*a*) Describe all statistical methods, including those used to control for confounding | 8-11 |
|  |  | (*b*) Describe any methods used to examine subgroups and interactions | 11 |
|  |  | (*c*) Explain how missing data were addressed | 6, 9 eFigure 1 |
|  |  | (*d*) If applicable, explain how loss to follow-up was addressed | 9-10 |
|  |  | (*e*) Describe any sensitivity analyses | 11 |
| Results | | |  |
| Participants | 13* | (a) Report numbers of individuals at each stage of study—eg numbers potentially eligible, examined for eligibility, confirmed eligible, included in the study, completing follow-up, and analysed | 6 eFigure 2 |
|  |  | (b) Give reasons for non-participation at each stage | eFigure 2 |
|  |  | (c) Consider use of a flow diagram | eFigure 2 |
| Descriptive data | 14* | (a) Give characteristics of study participants (eg demographic, clinical, social) and information on exposures and potential confounders | 11 Table 1 |
|  |  | (b) Indicate number of participants with missing data for each variable of interest | n/a |
|  |  | (c) Summarise follow-up time (eg, average and total amount) | 12 |
| Outcome data | 15* | Report numbers of outcome events or summary measures over time | 12-13 Table 2 |
| Main results | 16 | (*a*) Give unadjusted estimates and, if applicable, confounder-adjusted estimates and their precision (eg, 95% confidence interval). Make clear which confounders were adjusted for and why they were included | 12-13 Table 2 |
|  |  | (*b*) Report category boundaries when continuous variables were categorized | Table 1 Table 2 |
|  |  | (*c*) If relevant, consider translating estimates of relative risk into absolute risk for a meaningful time period | n/a |
| Other analyses | 17 | Report other analyses done—eg analyses of subgroups and interactions, and sensitivity analyses | 13  eTable 3-4 |
| Discussion |  |  |  |
| Key results | 18 | Summarise key results with reference to study objectives | 13 |
| Limitations | 19 | Discuss limitations of the study, taking into account sources of potential bias or imprecision. Discuss both direction and magnitude of any potential bias | 15-16 |
| Interpretation | 20 | Give a cautious overall interpretation of results considering objectives, limitations, multiplicity of analyses, results from similar studies, and other relevant evidence | 13-15 |
| Generalisability | 21 | Discuss the generalisability (external validity) of the study results | 16 |
| Other information |  |  |  |
| Funding | 22 | Give the source of funding and the role of the funders for the present study and, if applicable, for the original study on which the present article is based | 17 |

## eTable 2. Covariates used for the statistical models.

| **Variable** | **Variable Type** | **Covariate included in baseline treatment model** | **Covariates included for time-varying adherence and censoring models** | |
| --- | --- | --- | --- | --- |
|  |  |  | **Baseline value present in model and is not updated monthly** | **Value is updated monthly** |
| Age (years) | Continuous | X | X |  |
| Female | Dichotomous | X | X |  |
| Race and Ethnicity | Categorical, 4 levels: Non-Hispanic White (Referent), Non-Hispanic Black, Hispanic, and All other | X | X |  |
| Education status | Categorical, 4 levels: Less than high school (Referent), high school graduate only, post-high school graduate, and college graduate | X | X |  |
| Has health insurance | Dichotomous | X | X |  |
| Usual Source of Care, n (%) | Categorical, 3 levels: doctors’ office/outpatient clinic (Referent), community healthcare facility/other, no usual source of care | X | X |  |
| Clinical CVD | Dichotomous | X | X |  |
| History of atrial fibrillation | Dichotomous | X | X |  |
| History of depression | Dichotomous | X | X |  |
| Montreal Cognitive Assessment,* median (IQR) | Continuous | X |  | X |
| Logical Memory form II,^†^ median (IQR) | Continuous | X |  | X |
| Digit Symbol Coding Test,^‡^ median (IQR) | Continuous | X |  | X |
| Systolic BP (mm Hg) | Continuous | X |  | X |
| Diastolic BP (mm Hg) | Continuous | X | X |  |
| Resting heart rate (beats/minute) | Continuous | X | X |  |
| Serum potassium (mg/dL) | Continuous | X |  | X |
| Serum creatinine (mg/dL) | Continuous | X |  | X |
| Total cholesterol (mg/dL) | Continuous | X | X |  |
| HDL cholesterol (mg/dL) | Continuous | X | X |  |
| Triglycerides (mg/dL) | Continuous | X | X |  |
| Body mass index (kg/m^2^) | Continuous | X |  | X |
| Serum glucose (mg/dL) | Continuous |  |  | X |
| Aspirin use | Dichotomous | X | X |  |
| Statin use | Dichotomous | X | X |  |
| NSAID use | Dichotomous | X | X |  |
| Number of non-antihypertensive medications used | Continuous | X | X |  |
| Number of antihypertensive medications used | Continuous | X | X |  |
| ACEI use | Dichotomous | -- |  | X |
| ARB use | Dichotomous | -- |  | X |
| CCB use | Dichotomous | X |  | X |
| Thiazide diuretic use | Dichotomous | X |  | X |
| Loop diuretic use | Dichotomous | X |  | X |
| Beta-blocker use | Dichotomous | X |  | X |
| Alpha-blocker use | Dichotomous | X |  | X |
| Other antihypertensive sub-class use | Dichotomous | X |  | X |
| Intensive Treatment Arm | Dichotomous | X | X |  |
| Follow-up month | Continuous | -- |  | X |

Numbers are number (%), plus–minus values are means ± standard deviation, or square brackets are median [IQR].

*Scores range from 0 to 30, with higher scores denoting better cognitive function.

†Subtest of the Wechsler Memory Scale. Scores range from 0 to 14, with higher scores denoting better cognitive function.

‡Subtest of the Wechsler Adult Intelligence Scale. Scores range from 0 to 135, with higher scores denoting better cognitive function.

ACEI: angiotensin-converting enzyme inhibitor; ARB: angiotensin-II receptor blocker; CCB: calcium channel blocker; CVD: cardiovascular disease; BP: blood pressure; HDL: high-density lipoprotein; NSAID: non-steroidal anti-inflammatory drug; SPRINT: Systolic Blood Pressure Intervention Trial

## eTable 3. Reasons for censoring at each timepoint.

| Treatment group, censoring reason | Cumulative number lost by each follow-up month | | | | | | |
| --- | --- | --- | --- | --- | --- | --- | --- |
|  | 12 | 24 | 36 | 48 | 60 | 72 | 84 |
| ACEI |  |  |  |  |  |  |  |
| Discontinuation of initial treatment strategy | 570 | 718 | 800 | 849 | 854 | 854 | 854 |
| Loss to follow-up | 14 | 21 | 38 | 122 | 198 | 317 | 390 |
| Death | 5 | 14 | 20 | 26 | 29 | 29 | 29 |
| Total | 589 | 753 | 858 | 997 | 1081 | 1201 | 1273 |
| ARB |  |  |  |  |  |  |  |
| Discontinuation of initial treatment strategy | 157 | 223 | 263 | 286 | 290 | 290 | 290 |
| Loss to follow-up | 17 | 23 | 36 | 114 | 195 | 311 | 403 |
| Death | 1 | 5 | 6 | 8 | 10 | 10 | 10 |
| Total | 175 | 251 | 305 | 408 | 495 | 611 | 703 |

The table displays the number of participants lost to follow-up by each follow-up month, cumulatively. For example, 570 patients who initiated the ACEI protocol on their index date deviated from their assigned protocol by month 12.

ACEI: angiotensin-converting enzyme inhibitor; ARB: angiotensin-II receptor blocker

## eTable 4. Primary composite outcome results among subgroups.

| **Subgroup** | **Unadjusted (ie, Crude)** | | | **IP-weighted ITT analysis** | | | **IP-weighted per-protocol** | | | **P_interaction_** |
| --- | --- | --- | --- | --- | --- | --- | --- | --- | --- | --- |
|  | **Absolute risk at 4 years**  **(95% CI)** | | **4-year Risk Ratio**  **(95% CI)** | **Absolute risk at 4 years**  **(95% CI)** | | **4-year Risk Ratio**  **(95% CI)** | **Absolute risk at 4 years**  **(95% CI)** | | **4-year Risk Ratio**  **(95% CI)** |  |
|  | **ARB protocol**  **(N=710)** | **ACEI protocol**  **(N=1,289)** |  | **ARB protocol** | **ACEI protocol** |  | **ARB protocol** | **ACEI protocol** |  |  |
| **Amnestic MCI or PD** |  |  |  |  |  |  |  |  |  |  |
| Age, years |  |  |  |  |  |  |  |  |  |  |
| <75 | 0.10 (0.07,0.12) | 0.11 (0.09,0.12) | 0.91 (0.66,1.23) | 0.10 (0.08,0.14) | 0.10 (0.08,0.12) | 1.06 (0.76,1.45) | 0.09 (0.06,0.13) | 0.09 (0.06,0.12) | 1.04 (0.62,1.63) | 0.47 |
| ≥75 | 0.24 (0.18,0.31) | 0.30 (0.25,0.36) | 0.80 (0.57,1.11) | 0.27 (0.21,0.35) | 0.30 (0.24,0.35) | 0.92 (0.66,1.29) | 0.28 (0.18,0.38) | 0.36 (0.26,0.46) | 0.80 (0.48,1.23) | - |
| Sex |  |  |  |  |  |  |  |  |  |  |
| Male | 0.15 (0.12,0.19) | 0.16 (0.14,0.19) | 0.94 (0.70,1.24) | 0.17 (0.13,0.21) | 0.16 (0.14,0.18) | 1.05 (0.78,1.37) | 0.14 (0.09,0.20) | 0.17 (0.13,0.21) | 0.86 (0.52,1.34) | 0.59 |
| Female | 0.11 (0.08,0.15) | 0.13 (0.08,0.19) | 0.83 (0.53,1.22) | 0.12 (0.08,0.16) | 0.13 (0.08,0.19) | 0.94 (0.62,1.36) | 0.15 (0.09,0.21) | 0.13 (0.08,0.19) | 1.11 (0.56,2.07) | - |
| Race-ethnicity |  |  |  |  |  |  |  |  |  |  |
| Non-Hispanic Black | 0.17 (0.12,0.22) | 0.18 (0.14,0.22) | 0.95 (0.65,1.36) | 0.19 (0.14,0.25) | 0.17 (0.13,0.21) | 1.14 (0.79,1.61) | 0.21 (0.13,0.29) | 0.17 (0.10,0.23) | 1.26 (0.70,2.19) | 0.28 |
| All other race-ethnicities | 0.12 (0.09,0.15) | 0.14 (0.12,0.17) | 0.81 (0.59,1.08) | 0.13 (0.10,0.17) | 0.14 (0.12,0.16) | 0.94 (0.67,1.25) | 0.12 (0.07,0.17) | 0.15 (0.11,0.19) | 0.79 (0.47,1.22) | - |
| History of cardiovascular disease |  |  |  |  |  |  |  |  |  |  |
| Yes | 0.13 (0.08,0.20) | 0.17 (0.13,0.22) | 0.78 (0.41,1.27) | 0.16 (0.09,0.25) | 0.16 (0.12,0.21) | 0.98 (0.54,1.61) | 0.10 (0.04,0.18) | 0.22 (0.14,0.32) | 0.46 (0.17,0.99) | 0.03 |
| No | 0.14 (0.11,0.16) | 0.15 (0.13,0.17) | 0.91 (0.70,1.15) | 0.15 (0.12,0.18) | 0.14 (0.12,0.17) | 1.03 (0.79,1.30) | 0.15 (0.11,0.20) | 0.14 (0.11,0.17) | 1.10 (0.74,1.61) | - |
| Randomization arm |  |  |  |  |  |  |  |  |  |  |
| Intensive | 0.16 (0.11,0.21) | 0.15 (0.12,0.19) | 1.06 (0.68,1.54) | 0.16 (0.11,0.21) | 0.15 (0.12,0.19) | 1.06 (0.68,1.54) | 0.16 (0.11,0.21) | 0.15 (0.12,0.19) | 1.06 (0.68,1.54) | 0.43 |
| Standard | 0.12 (0.05,0.20) | 0.16 (0.10,0.21) | 0.75 (0.32,1.50) | 0.12 (0.05,0.20) | 0.16 (0.10,0.21) | 0.75 (0.32,1.50) | 0.12 (0.05,0.20) | 0.16 (0.10,0.21) | 0.75 (0.32,1.50) | - |
| **Amnestic MCI, PD, or death** |  |  |  |  |  |  |  |  |  |  |
| Age, years |  |  |  |  |  |  |  |  |  |  |
| <75 | 0.11 (0.08,0.14) | 0.15 (0.13,0.17) | 0.91 (0.56,0.97) | 0.12 (0.09,0.15) | 0.14 (0.12,0.16) | 0.85 (0.64,1.14) | 0.10 (0.07,0.14) | 0.13 (0.10,0.17) | 0.80 (0.52,1.22) | 0.7 |
| ≥75 | 0.28 (0.22,0.35) | 0.39 (0.33,0.44) | 0.80 (0.54,0.95) | 0.31 (0.24,0.39) | 0.38 (0.32,0.43) | 0.83 (0.62,1.09) | 0.31 (0.21,0.42) | 0.44 (0.34,0.56) | 0.72 (0.44,1.03) | - |
| Sex |  |  |  |  |  |  |  |  |  |  |
| Male | 0.18 (0.14,0.21) | 0.22 (0.20,0.25) | 0.94 (0.60,1.01) | 0.19 (0.15,0.23) | 0.22 (0.19,0.25) | 0.86 (0.67,1.10) | 0.16 (0.11,0.22) | 0.22 (0.18,0.27) | 0.73 (0.48,1.05) | 0.54 |
| Female | 0.13 (0.09,0.17) | 0.17 (0.14,0.21) | 0.83 (0.52,1.06) | 0.14 (0.10,0.18) | 0.16 (0.13,0.20) | 0.86 (0.60,1.21) | 0.16 (0.10,0.23) | 0.18 (0.12,0.24) | 0.91 (0.52,1.52) | - |
| Race-ethnicity |  |  |  |  |  |  |  |  |  |  |
| Non-Hispanic Black | 0.18 (0.13,0.24) | 0.24 (0.20,0.29) | 0.95 (0.53,1.04) | 0.20 (0.15,0.26) | 0.23 (0.19,0.27) | 0.89 (0.63,1.21) | 0.22 (0.13,0.30) | 0.26 (0.19,0.34) | 0.84 (0.48,1.28) | 0.8 |
| All other race-ethnicities | 0.14 (0.11,0.18) | 0.19 (0.17,0.22) | 0.81 (0.56,0.98) | 0.16 (0.12,0.20) | 0.19 (0.17,0.22) | 0.85 (0.65,1.09) | 0.14 (0.09,0.19) | 0.18 (0.14,0.22) | 0.77 (0.49,1.14) | - |
| History of cardiovascular disease |  |  |  |  |  |  |  |  |  |  |
| Yes | 0.18 (0.12,0.26) | 0.27 (0.21,0.33) | 0.78 (0.41,1.00) | 0.20 (0.13,0.29) | 0.26 (0.20,0.32) | 0.79 (0.48,1.19) | 0.14 (0.07,0.22) | 0.31 (0.21,0.42) | 0.44 (0.21,0.80) | 0.04 |
| No | 0.15 (0.12,0.18) | 0.19 (0.17,0.22) | 0.91 (0.63,0.98) | 0.17 (0.13,0.20) | 0.19 (0.16,0.21) | 0.89 (0.70,1.09) | 0.17 (0.12,0.22) | 0.18 (0.15,0.22) | 0.91 (0.64,1.25) | - |
| Randomization arm |  |  |  |  |  |  |  |  |  |  |
| Intensive | 0.17 (0.12,0.23) | 0.19 (0.15,0.24) | 1.06 (0.62,1.27) | 0.17 (0.12,0.23) | 0.19 (0.15,0.24) | 0.91 (0.62,1.27) | 0.17 (0.12,0.23) | 0.19 (0.15,0.24) | 0.91 (0.62,1.27) | 0.29 |
| Standard | 0.14 (0.07,0.22) | 0.22 (0.16,0.29) | 0.75 (0.30,1.12) | 0.14 (0.07,0.22) | 0.22 (0.16,0.29) | 0.62 (0.30,1.12) | 0.14 (0.07,0.22) | 0.22 (0.16,0.29) | 0.62 (0.30,1.12) | - |
| **All-cause death** |  |  |  |  |  |  |  |  |  |  |
| Age, years |  |  |  |  |  |  |  |  |  |  |
| <75 | 0.02 (0.01,0.03) | 0.04 (0.03,0.06) | 0.36 (0.12,0.71) | 0.01 (0.00,0.03) | 0.04 (0.03,0.05) | 0.36 (0.12,0.70) | 0.02 (0.00,0.03) | 0.04 (0.02,0.06) | 0.38 (0.04,0.94) | 0.82 |
| ≥75 | 0.04 (0.02,0.08) | 0.11 (0.07,0.14) | 0.40 (0.15,0.79) | 0.04 (0.01,0.08) | 0.11 (0.07,0.14) | 0.40 (0.13,0.80) | 0.03 (0.00,0.06) | 0.09 (0.04,0.16) | 0.30 (0.05,1.10) | - |
| Sex |  |  |  |  |  |  |  |  |  |  |
| Male | 0.02 (0.01,0.04) | 0.07 (0.05,0.09) | 0.35 (0.14,0.62) | 0.02 (0.01,0.04) | 0.07 (0.06,0.09) | 0.33 (0.13,0.59) | 0.02 (0.00,0.03) | 0.06 (0.03,0.09) | 0.34 (0.07,0.72) | 0.95 |
| Female | 0.02 (0.00,0.05) | 0.03 (0.02,0.05) | 0.74 (0.15,1.86) | 0.02 (0.00,0.04) | 0.03 (0.01,0.05) | 0.78 (0.07,2.05) | 0.02 (0.00,0.05) | 0.04 (0.01,0.08) | 0.47 (0.00,2.27) | - |
| Race-ethnicity |  |  |  |  |  |  |  |  |  |  |
| Non-Hispanic Black | 0.01 (0.00,0.03) | 0.08 (0.05,0.11) | 0.19 (0.00,0.44) | 0.01 (0.00,0.02) | 0.07 (0.05,0.10) | 0.16 (0.00,0.38) | 0.01 (0.00,0.02) | 0.09 (0.04,0.15) | 0.09 (0.00,0.26) | 0.14 |
| All other race-ethnicities | 0.03 (0.01,0.04) | 0.05 (0.04,0.07) | 0.52 (0.24,0.94) | 0.03 (0.01,0.04) | 0.05 (0.04,0.07) | 0.52 (0.23,0.92) | 0.02 (0.01,0.04) | 0.03 (0.02,0.06) | 0.65 (0.20,1.60) | - |
| History of cardiovascular disease |  |  |  |  |  |  |  |  |  |  |
| Yes | 0.05 (0.01,0.10) | 0.12 (0.08,0.16) | 0.46 (0.10,0.96) | 0.05 (0.01,0.09) | 0.11 (0.07,0.16) | 0.43 (0.09,0.91) | 0.05 (0.01,0.09) | 0.12 (0.05,0.20) | 0.40 (0.04,1.30) | 0.84 |
| No | 0.02 (0.01,0.03) | 0.04 (0.03,0.06) | 0.39 (0.16,0.69) | 0.02 (0.01,0.03) | 0.05 (0.03,0.06) | 0.38 (0.15,0.68) | 0.01 (0.00,0.03) | 0.04 (0.02,0.06) | 0.31 (0.04,0.79) | - |
| Randomization arm |  |  |  |  |  |  |  |  |  |  |
| Intensive | 0.02 (0.00,0.04) | 0.06 (0.03,0.09) | 0.34 (0.08,0.85) | 0.02 (0.00,0.04) | 0.06 (0.03,0.09) | 0.34 (0.08,0.85) | 0.02 (0.00,0.04) | 0.06 (0.03,0.09) | 0.34 (0.08,0.85) | 0.94 |
| Standard | 0.02 (0.00,0.04) | 0.05 (0.02,0.08) | 0.38 (0.00,1.47) | 0.02 (0.00,0.04) | 0.05 (0.02,0.08) | 0.38 (0.00,1.47) | 0.02 (0.00,0.04) | 0.05 (0.02,0.08) | 0.38 (0.00,1.47) | - |

ACEI: angiotensin-converting enzyme inhibitor; ARB: angiotensin-II receptor blocker; CI: confidence interval; IP: inverse probability; MCI: mild cognitive impairment; PD: probable dementia

## eTable 5. Sensitivity analyses on the primary outcome.

| **Outcome** | **Unadjusted (i.e., Crude)** | | | **IP-weighted ITT analysis** | | | **IP-weighted per-protocol** | | |
| --- | --- | --- | --- | --- | --- | --- | --- | --- | --- |
|  | **Absolute risk at 4 years**  **(95% CI)** | | **4-year Risk Ratio**  **(95% CI)** | **Absolute risk at 4 years**  **(95% CI)** | | **4-year Risk Ratio**  **(95% CI)** | **Absolute risk at 4 years**  **(95% CI)** | | **4-year Risk Ratio**  **(95% CI)** |
|  | **ARB protocol**  **(N=710)** | **ACEI protocol**  **(N=1,289)** |  | **ARB protocol** | **ACEI protocol** |  | **ARB protocol** | **ACEI protocol** |  |
| **Amnestic MCI or PD** |  |  |  |  |  |  |  |  |  |
| Expand initiation window to 24 months | 0.14 (0.12,0.17) | 0.16 (0.15,0.18) | 0.86 (0.70,1.04) | 0.16 (0.13,0.19) | 0.16 (0.14,0.18) | 1.01 (0.83,1.19) | 0.16 (0.12,0.20) | 0.16 (0.14,0.20) | 0.95 (0.71,1.29) |
| Treatment-related SAE counts as protocol deviation | 0.14 (0.11,0.16) | 0.15 (0.13,0.17) | 0.88 (0.70,1.10) | 0.15 (0.12,0.18) | 0.15 (0.13,0.17) | 1.01 (0.81,1.24) | 0.14 (0.10,0.18) | 0.15 (0.12,0.19) | 0.94 (0.66,1.30) |
| **Amnestic MCI, PD, or death** |  |  |  |  |  |  |  |  |  |
| Expand initiation window to 24 months | 0.16 (0.14,0.19) | 0.21 (0.19,0.23) | 0.77 (0.64,0.92) | 0.18 (0.15,0.21) | 0.20 (0.18,0.22) | 0.89 (0.74,1.04) | 0.18 (0.14,0.22) | 0.21 (0.18,0.25) | 0.83 (0.63,1.06) |
| Treatment-related SAE counts as protocol deviation | 0.16 (0.13,0.18) | 0.21 (0.19,0.23) | 0.75 (0.61,0.92) | 0.17 (0.14,0.20) | 0.20 (0.18,0.22) | 0.86 (0.71,1.03) | 0.16 (0.12,0.20) | 0.20 (0.17,0.24) | 0.79 (0.58,1.05) |
| **All-cause death** |  |  |  |  |  |  |  |  |  |
| Expand initiation window to 24 months | 0.03 (0.02,0.04) | 0.06 (0.05,0.07) | 0.45 (0.27,0.70) | 0.03 (0.02,0.04) | 0.06 (0.04,0.07) | 0.46 (0.26,0.73) | 0.03 (0.01,0.05) | 0.05 (0.03,0.07) | 0.49 (0.22,0.98) |
| Treatment-related SAE counts as protocol deviation | 0.02 (0.01,0.03) | 0.06 (0.05,0.07) | 0.39 (0.21,0.62) | 0.02 (0.01,0.03) | 0.06 (0.05,0.07) | 0.39 (0.20,0.63) | 0.02 (0.01,0.03) | 0.05 (0.03,0.08) | 0.36 (0.14,0.76) |

ACEI: angiotensin-converting enzyme inhibitor; ARB: angiotensin-II receptor blocker; CI: confidence interval; IP: inverse probability; MCI: mild cognitive impairment; PD: probable dementia; SAE: serious adverse event

# eFigures

## eFigure 1. Study design schema.


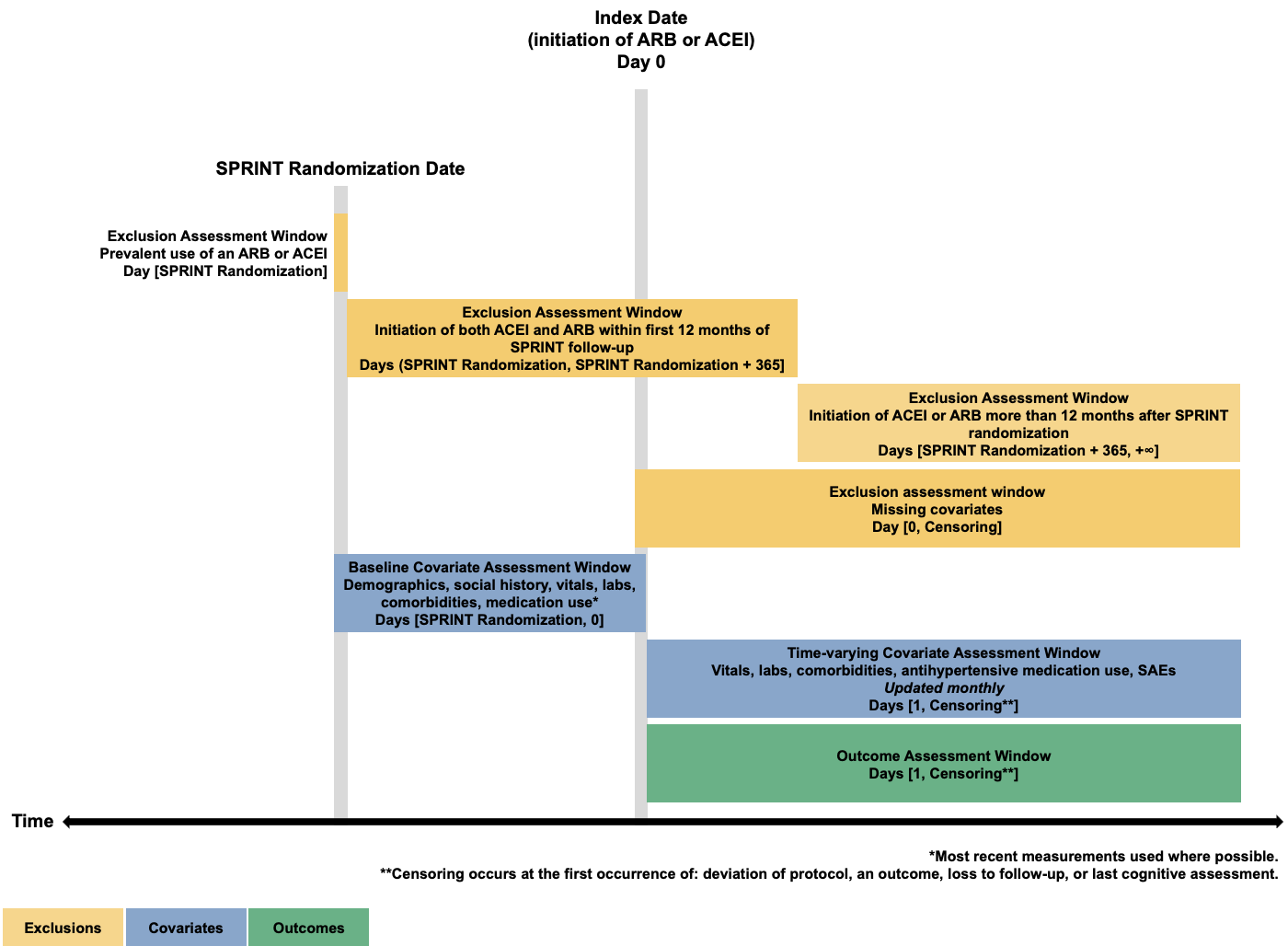


Participants could be followed for a minimum of 1 day up to a maximum of 2,689 days (89.6 months; i.e., the time period between randomization and the final study close-out date). *Abbreviations:* ACEI: angiotensin-converting enzyme inhibitor; ARB: angiotensin-II receptor blocker; SAE: serious adverse event; SPRINT: Systolic Blood Pressure Intervention Trial

## eFigure 2. Flowchart for inclusion in the current study.


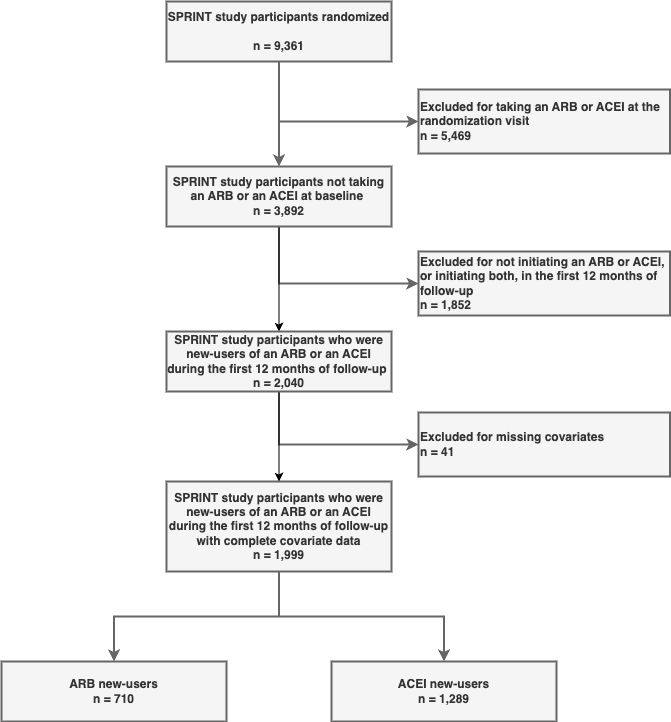


*Abbreviations:* ACEI: angiotensin-converting enzyme inhibitor; ARB: angiotensin-II receptor blocker; SPRINT: Systolic Blood Pressure Intervention Trial

## eFigure 3. Directed acyclic graph describing the proposed relationships between the exposure, covariates, and outcomes.


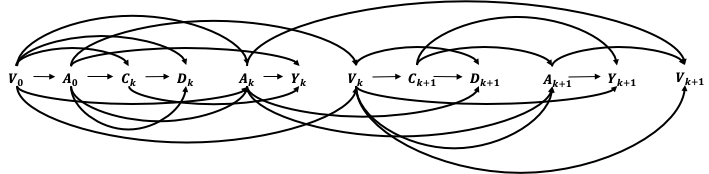


Let $\boldsymbol{V}_{\boldsymbol{0}}$ be pre-initiation covariates and $\boldsymbol{A}_{\boldsymbol{0}}$ be an indicator for initiation of ARB (ACEI) protocol at baseline/index month. Also, let $\boldsymbol{A}_{\boldsymbol{k}}$ indicate whether the patient adhered to treatment protocol during month $\boldsymbol{k}$. Let $\boldsymbol{C}_{\boldsymbol{k}}$ indicate whether the patient has been lost to follow-up by the end of month $\boldsymbol{k}$. Let $\boldsymbol{D}_{\boldsymbol{k}}$ be an indicator of death during month $\boldsymbol{k}$. Let $\boldsymbol{Y}_{\boldsymbol{k}}$ be an indicator of non-terminal outcome (e.g., probable dementia) during month $\boldsymbol{k}$. Let $\boldsymbol{V}_{\boldsymbol{k}}$ be a vector of covariates scheduled to be measured in month $\boldsymbol{k}$. With these definitions, the data for an individual during month $\boldsymbol{k}$ are observed in the following temporal order: $\boldsymbol{O}_{\boldsymbol{k}}=$ $\left( \boldsymbol{C}_{\boldsymbol{k}},\boldsymbol{D}_{\boldsymbol{k}},\boldsymbol{A}_{\boldsymbol{k}},\boldsymbol{Y}_{\boldsymbol{k}},\boldsymbol{V}_{\boldsymbol{k}} \right)$.

$\boldsymbol{V}_{\boldsymbol{0}}$ variables included sociodemographic characteristics (including self-reported race and ethnicity), comorbidities, cognitive function, and concomitant non-antihypertensive medication use, systolic BP, diastolic BP, laboratory measurements, and non-ARB or -ACEI antihypertensive medication use.

$\boldsymbol{V}_{\boldsymbol{k}}$ variables included use of all other antihypertensive classes; systolic BP; body mass index; and serum potassium, creatinine, and glucose; and all cognitive tests (Montreal Cognitive Assessment, Logical Memory form II, Digit Symbol Coding Test. ß

## eFigure 4. Distribution of probability of baseline ARB initiation among ARB initiators (bottom panel) and ACEI initiators (top panel).


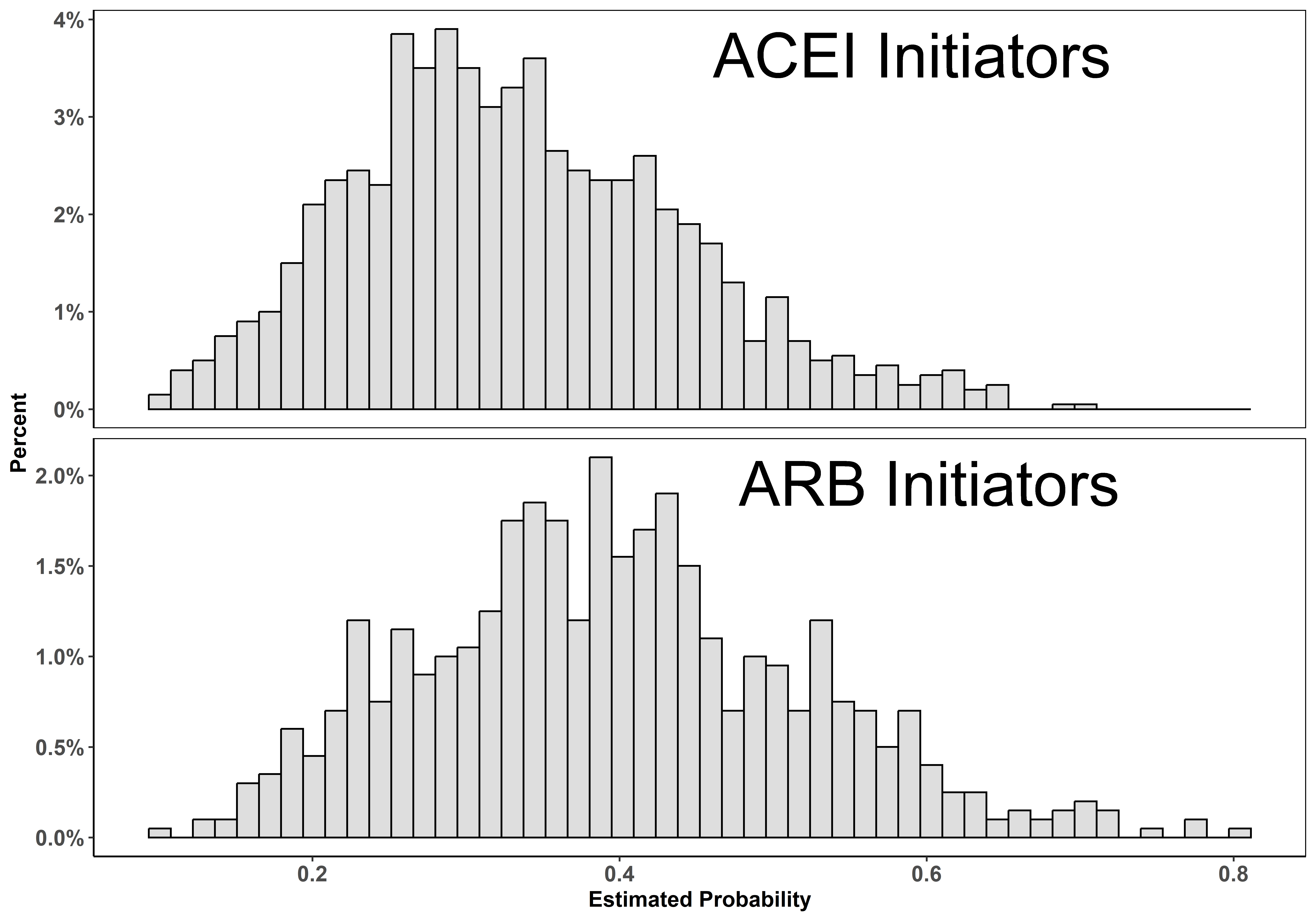


*Abbreviations:* ACEI: angiotensin-converting enzyme inhibitor; ARB: angiotensin-II receptor blocker; SPRINT: Systolic Blood Pressure Intervention Trial

## eFigure 5. Distribution of stabilized inverse probability weights for ARB vs. ACEI treatment across follow-up.





*Abbreviations:* ACEI: angiotensin-converting enzyme inhibitor; ARB: angiotensin-II receptor blocker; SPRINT: Systolic Blood Pressure Intervention Trial

## eFigure 6. Balance of patient characteristics before and after inverse propensity score weighting at baseline.


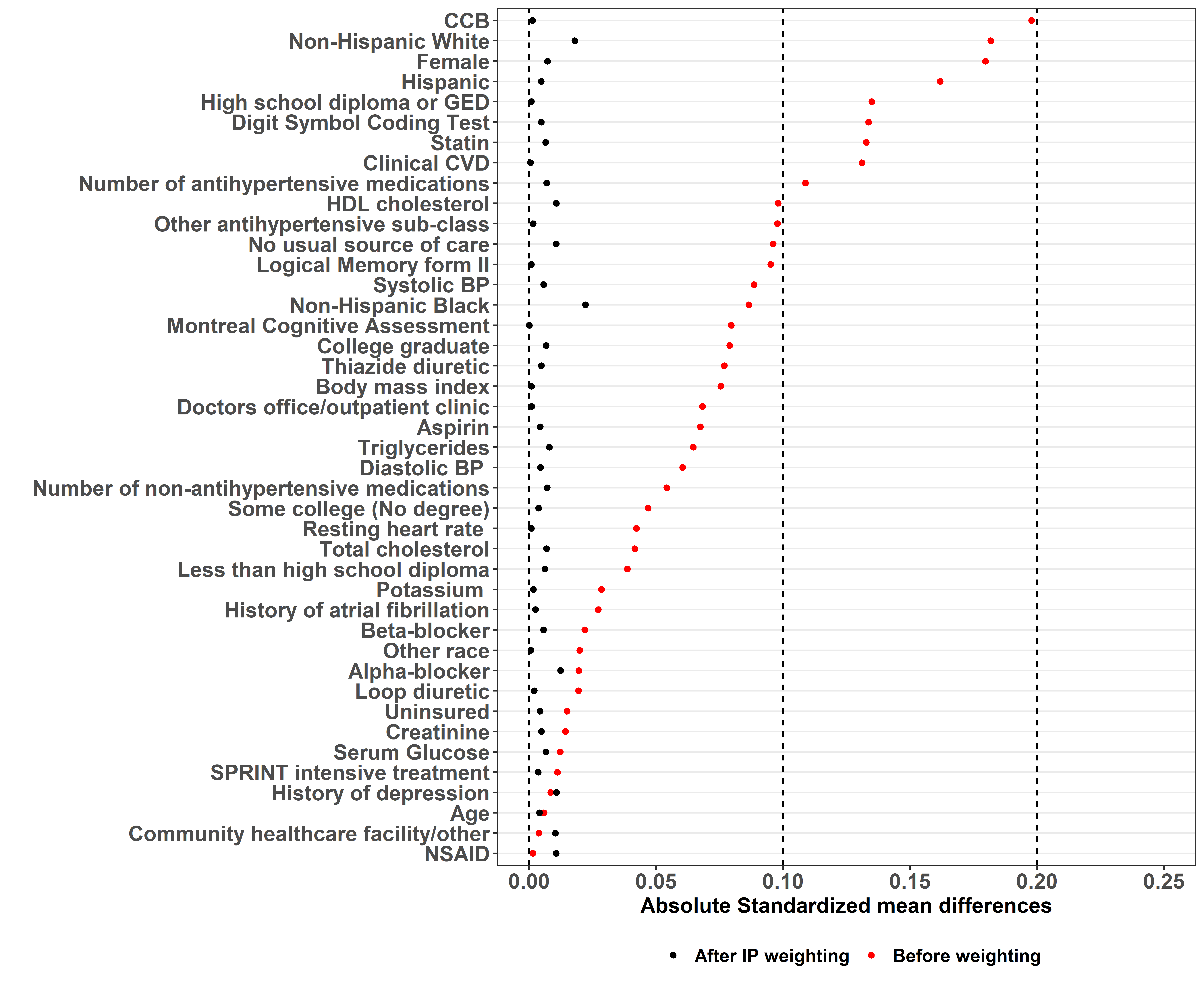


Red circles represent the ASMD between the ARB and ACEI groups before weighting; black circles represent the ASMD after weighting.

*Abbreviations:* ACEI: angiotensin-converting enzyme inhibitor; ARB: angiotensin-II receptor blocker; CCB: calcium channel blocker; CVD: cardiovascular disease; BP: blood pressure; HDL: high-density lipoprotein; NSAID: non-steroidal anti-inflammatory drug; SPRINT: Systolic Blood Pressure Intervention Trial

## eFigure 7. Balance of patient characteristics before and after applying time-varying adherence weights within ARB initiators (Panel A) and ACEI initiators (Panel B).

Panel A: ARB initiators

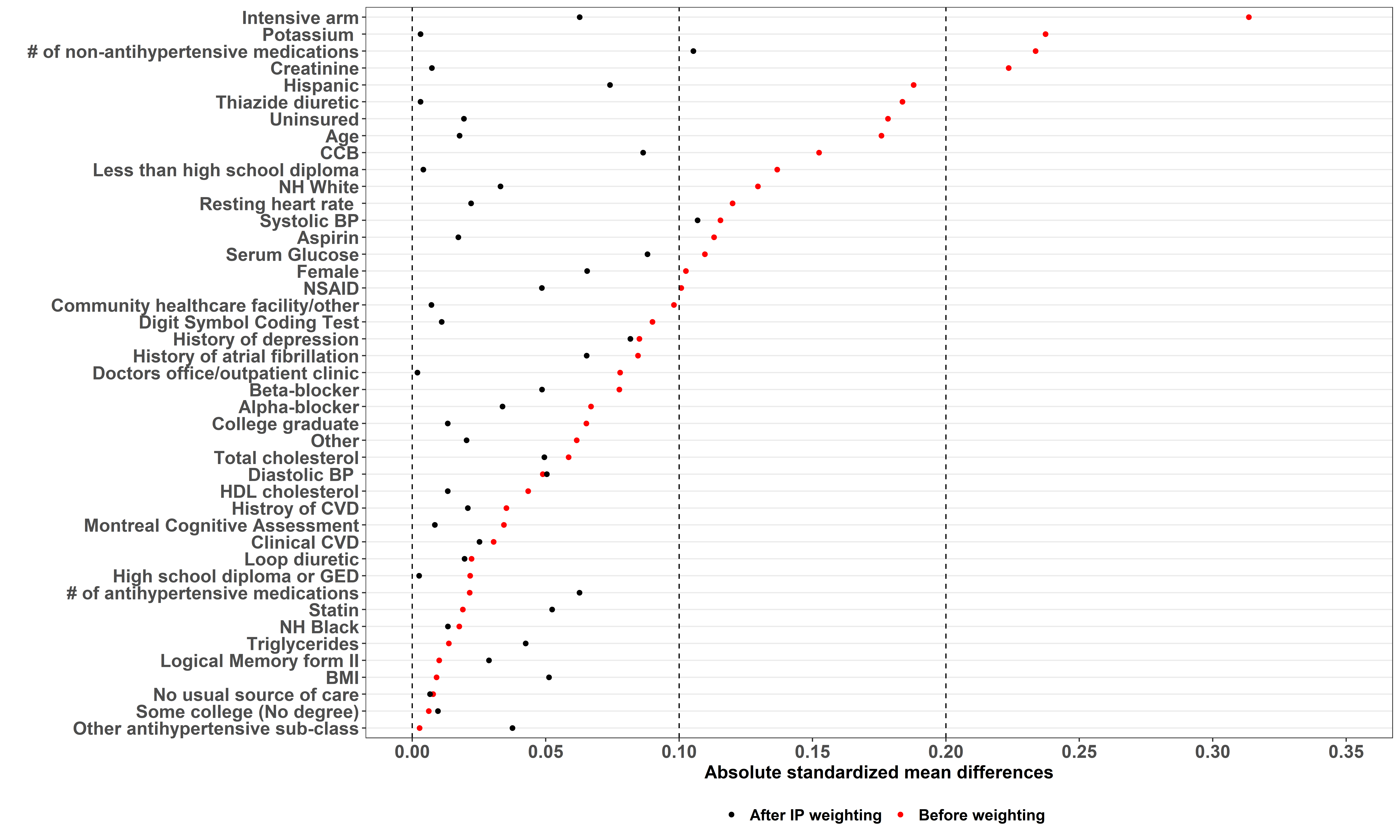


Panel B: ACEI initiators

**
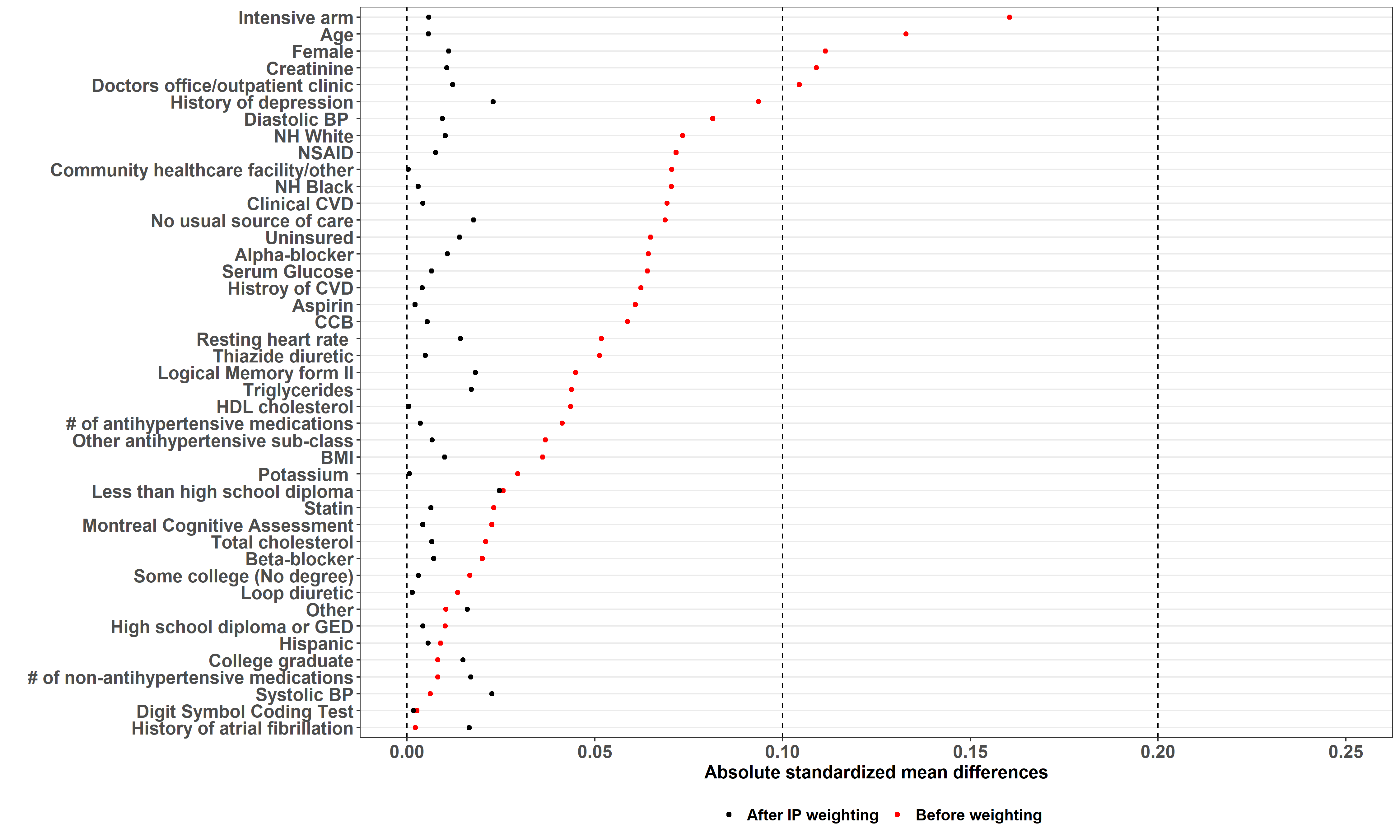
**

*Abbreviations:* ACEI: angiotensin-converting enzyme inhibitor; ARB: angiotensin-II receptor blocker; CCB: calcium channel blocker; CVD: cardiovascular disease; BP: blood pressure; HDL: high-density lipoprotein; NSAID: non-steroidal anti-inflammatory drug; SPRINT: Systolic Blood Pressure Intervention Trial

## eFigure 8. Balance of patient characteristics before and after applying time-varying censoring weights within ARB initiators (Panel A) and ACEI initiators (Panel B).

Panel A: ARB initiators

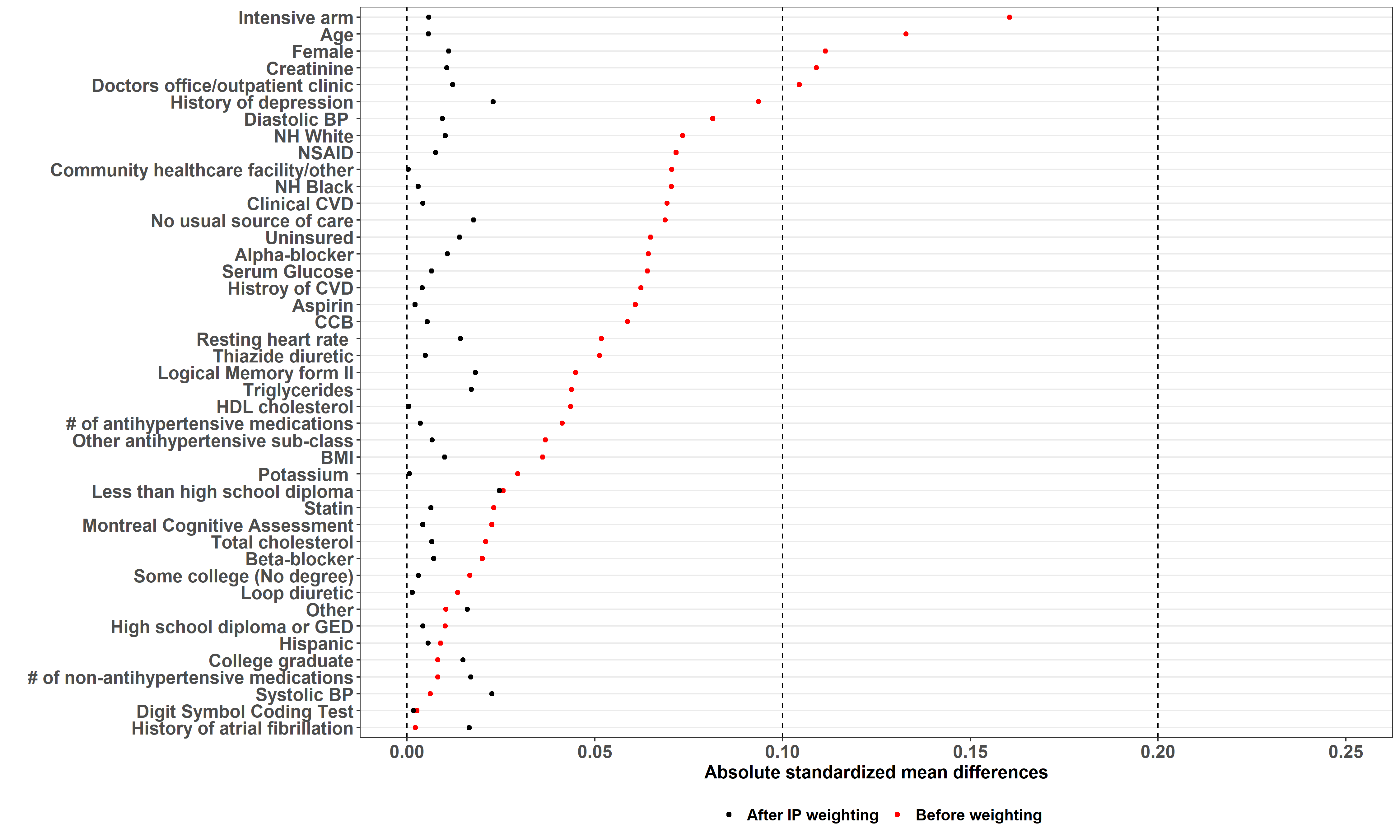


Panel B: ACEI initiators


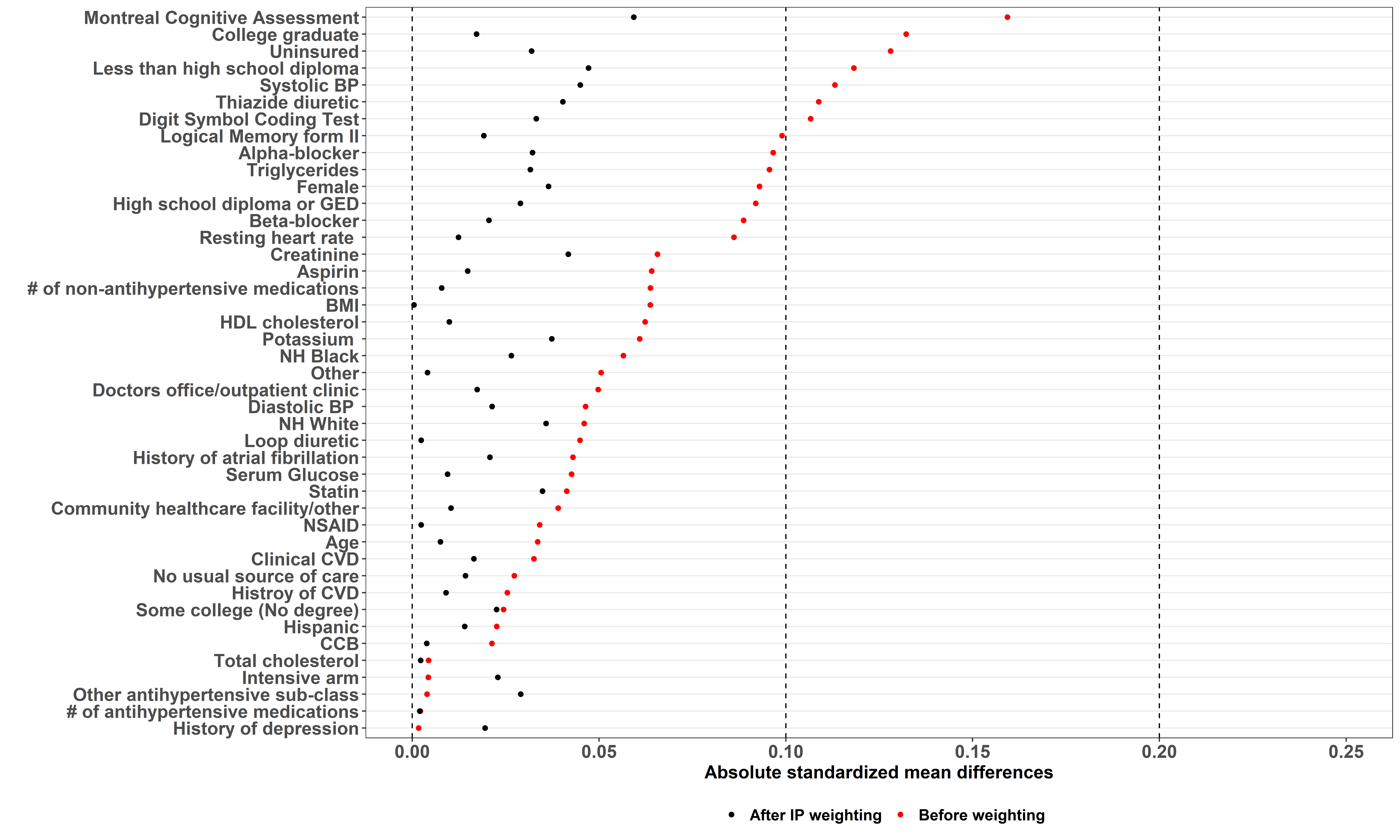


*Abbreviations:* ACEI: angiotensin-converting enzyme inhibitor; ARB: angiotensin-II receptor blocker; CCB: calcium channel blocker; CVD: cardiovascular disease; BP: blood pressure; HDL: high-density lipoprotein; NSAID: non-steroidal anti-inflammatory drug; SPRINT: Systolic Blood Pressure Intervention Trial
